# Does cryptic microbiota mitigate pine resistance to an invasive beetle-fungus complex? Implications for invasion potential

**DOI:** 10.1038/srep33110

**Published:** 2016-09-13

**Authors:** Chihang Cheng, Letian Xu, Dandan Xu, Qiaozhe Lou, Min Lu, Jianghua Sun

**Affiliations:** 1 State Key Laboratory of Integrated Management of Pest Insects and Rodents, Institute of Zoology, Chinese Academy of Sciences, Beijing, 100101, China; 2 College of Life Sciences, Huzhou University, Huzhou, 313000, China; 3 TEDA Institute of Biological Sciences and Biotechnology, Nankai University, Tianjin, 300457, China; 4 University of Chinese Academy of Sciences, Beijing, 100049, China; 5 Technical Center, Hebei Entry-Exit Inspection and Quarantine Bureau, Shijiazhuang, 050051, China

## Abstract

Microbial symbionts are known to assist exotic pests in their colonization of new host plants. However, there has been little evidence linking symbiotic invasion success to mechanisms for mitigation of native plant resistance. The red turpentine beetle (RTB) was introduced with a fungus, *Leptographium procerum*, to China from the United States and became a destructively invasive symbiotic complex in natural *Pinus tabuliformis* forests. Here, we report that three Chinese-resident fungi, newly acquired by RTB in China, induce high levels of a phenolic defensive chemical, naringenin, in pines. This invasive beetle-fungus complex is suppressed by elevated levels of naringenin. However, cryptic microbiotas in RTB galleries strongly degrade naringenin, and pinitol, the main soluble carbohydrate of *P. tabuliformis*, is retained in *L. procerum*-infected phloem and facilitate naringenin biodegradation by the microbiotas. These results demonstrate that cryptic microbiota mitigates native host plant phenolic resistance to an invasive symbiotic complex, suggesting a putative mechanism for reduced biotic resistance to symbiotic invasion.

Successful single-species invasions have been attributed to reduced biotic resistance in native communities^1^,^2^. Especially where a series of strong, nested interactions exists, biotic resistance can become a highly important determinant in their invasions^3^,^4^ as suggested by the ‘new associations’ or ‘increased susceptibility’ hypotheses^5^,^6^. Recent research highlights several cases of pest invasions that are facilitated by symbiotic microbes^7^,^8^. Although this form of invasion seems successful, the limitation of invaders under condition of biotic resistance is suggested to be more severe if their interdependent microbial symbionts are quenched^9^,^10^. Symbiotic invasion might be more susceptible to biotic resistance than single-species invasions. However, there has been little evidence linking symbiotic invasion success to mechanisms for reduced biotic resistance.

Studies have reported that compromised plant defences have contributed to the success of invasive insect pests on their new hosts^11^,^12^,^13^. Even though it is considered as an important barrier for colonization, the role of plant chemical defence as mediated by an invader’s microbial symbionts has received little attention. For coniferous trees, the main products of chemical defence in the bark are terpenoid resins and phenolics^14^. Volatile terpenoids are known to serve as olfactory cues for host recognition, mediate tritrophic interactions and act as precursors for bark beetle pheromone signalling^15^,^16^,^17^. Non-volatile terpenoids like diterpene resin acids are suggested to mainly inhibit fungal associates with no obvious negative effects on bark beetles^18^. Phenolics are also abundant and chemically diverse (e.g. monoaryls, flavonoids and stilbenes) in conifer bark^19^,^20^,^21^, within which compounds of the stilbenes are reported to have both the antifeedant and antifungal properties to bark beetles and blue-staining fungal associates^22^,^23^. Bark beetles are commonly associated with ophiostomatoid fungi^24^ and also with diverse bacterial and yeast species^25^,^26^. Conifer defensive chemicals were shown to be regulated by either type of these microbial associates^27^,^28^,^29^,^30^,^31^, yet how these symbiotic partners jointly mediate the bark beetle-conifer defence interactions has been little evidenced empirically.

The red turpentine beetle (RTB), *Dendroctonus valens*, was accidently introduced into China from the United States about three decades ago and, following a latency period, suddenly became a widespread tree killer, devastating forests and killing over ten million healthy *Pinus tabuliformis* trees^32^. Female pioneer beetles initiate attacks, construct galleries and perform monogamous behaviour that one male is attracted by one female for reproduction^33^,^34^; and during these processes, ophiostomatoid fungal associates are inoculated into host pines. The fungal community of Chinese RTB contained 20 ophiostomatoid fungal species, great majority (15 species) of which were not found associated with the US RTB and are herein referred to “Chinese-resident” fungi. The rest of fungi were associated with RTB both in China and US: *Leptographium procerum* is the primary fungal associate of Chinese RTB, which had all alleles shared by US RTB-associated *L. procerum*, with no allele unique in Chinese populations, this demonstrating that *L. procerum* associated with RTB in China originated from those with RTB in US and thus is referred to as “Chinese-invasive”; the other species that had no population genetic evidence yet are temporarily classified as “shared” fungal species. Overall, the fungal community composition has shifted from species associated with RTB in the United States to species newly acquired in China (See Supplementary Table S1).

The multispecies interactions between RTB, ophiostomatoid fungi and host pine *P. tabuliformis* have been suggested to facilitate invasion success. After their introduction into China, *L. procerum* evolved several novel genotypes that were more pathogenic to host pines and induced the release of higher amounts of 3-carene, a volatile terpenoid which is the primary attractant for the beetle vector^35^,^36^. RTB and *L. procerum* act as an invasive symbiotic complex in China^32^. However, *L. procerum* could be outcompeted by newly acquired Chinese-resident fungi^37^. From the benefit of induced rosin defence by resident fungi, their suppression of *L. procerum* was largely alleviated in the presence of the non-volatile terpenoid, diterpene resin acid^37^. Phenolics have the potential in targeting both beetles and ophiostomatoid fungi, and in return, it is fascinating to know in here how this invasive beetle-fungus complex could protect itself against this type of defensive chemical in pine phloem. Thus in this study, we first investigated whether fungal associates of RTB in China differentially induced phenolic resistance by conducting fungal inoculations on *P. tabuliformis* seedlings. Then, we assessed *in vitro* whether performance of this invasive symbiotic complex would be affected under increased phenolic resistance. Last, we tested whether harmful phenolic compounds could be decreased by associated bacteria or yeasts, symbionts that may exist cryptically in RTB gallery, through phenolic biodegradation.

## Results

### High concentrations of naringenin induced in *P. tabuliformis* seedlings by Chinese-resident fungi associated with *D. valens*

**Figure 1:**
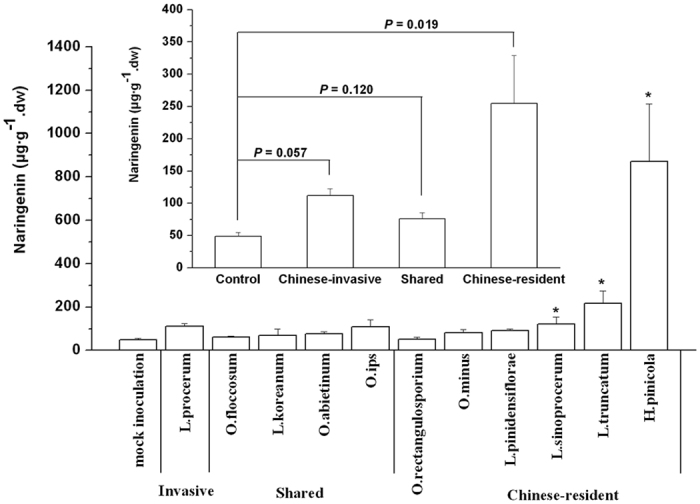
Among the ophiostomatoid fungal community, Chinese-resident fungi induce high concentrations of naringenin in pine seedlings. Mean concentrations of naringenin (+SEM, *n* = 3–5) in 4-5-yr-old *P. tabuliformis* seedlings were obtained from inoculation with the 11 fungal associates of Chinese RTB after 21 d. Asterisks indicate significant differences between controls and isolates (*P < 0.05). Inset: Mean concentrations of naringenin (+SEM) induced by the Chinese-invasive, shared, and Chinese-resident fungal groups.

Among the Chinese-invasive, shared, and Chinese-resident fungal groups, one phenolic compound of the flavonoids, naringenin, showed significant induction by the Chinese-resident group, compared to controls 21 d after inoculation (Fig. 1 inset; Kruskal-Wallis test, 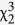 = 11.517, *P* < 0.01). Other phenolics including two monoaryls *ρ*-coumaric acid and ferulic acid, three stilbenes resveratrol, pinosylvin and pinosylvin monomethyl ether and another flavonoid taxifolin were not significantly induced by any fungal groups (Supplementary Table S2). Three Chinese-resident species, *H. pinicola*, *L. truncatum*, and *L. sinoprocerum*, among the representative 11 fungal species, induced significantly higher concentrations of naringenin compared to controls (Fig. 1; Kruskal-Wallis test, 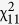 = 33.153, *P* < 0.001). The three Chinese-resident fungi and the Chinese-invasive *L. procerum* were then chosen for subsequent inoculation experiments.

**Figure 2:**
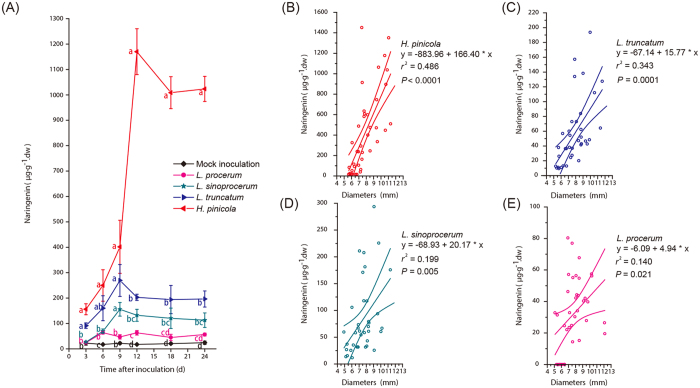
Three Chinese-resident fungi induce high concentrations of naringenin over time and across stem diameters of pine seedlings. (**A**) Mean concentrations of naringenin (±SEM, *n* = 7–10) from 4-5-yr-old *P. tabuliformis* seedlings inoculated with three Chinese-resident fungi and *L. procerum* after 3, 6, 9, 12, 18, and 24 d. Different letters indicate significant differences between treatments (*P* < 0.05). (**B–E**) Relationships between stem diameter and naringenin concentration in pine seedlings (*n* = 37–40) inoculated with three Chinese-resident fungi and *L. procerum* after 24 d.

There were significant differences in naringenin concentrations 3, 6, 9, 12, 18, and 24 d after inoculation among all the treatments (Fig. 2A; two-way ANOVA; isolate, *F*_4, 224_ = 300.756, *P* < 0.0001; time, *F*_5, 224_ = 39.359, *P* < 0.0001; isolate × time, *F*_20, 224_ = 26.799, *P* < 0.0001). *H. pinicola* and *L. truncatum* induced higher concentrations of naringenin than mock inoculation 3, 6, 9, 12, 18, and 24 d after inoculation and *L. sinoprocerum* induced higher concentrations of naringenin than mock inoculation 6, 9, 12, 18, and 24 d after inoculation (Fig. 2A). However, there were no significant differences in naringenin concentrations between *L. procerum* and mock inoculation at all the sampled points except 6 and 12 d after inoculation (Fig. 2A). Neither were there significant inductions in the concentrations of other phenolic compounds by any fungal associates compared to controls (Supplementary Figs S1–S7).

Diameter of *P. tabuliformis* seedlings was a significant predictor of naringenin induction for the four fungal associates (Fig. 2B–E; *H. pinicola*, *r*^*2*^ = 0.486, *F*_1, 38_ = 35.958, *P* < 0.0001; *L. truncatum*, *r*^*2*^ = 0.343, *F*_1, 35_ = 18.295, *P* = 0.0001; *L. sinoprocerum*, *r*^*2*^ = 0.199, *F*_1, 36_ = 8.922, *P* = 0.005; *L. procerum*, *r*^*2*^ = 0.140, *F*_1, 36_ = 5.854, *P* = 0.021). The standardized slopes of regression between *L. procerum* and each of the three Chinese-resident fungi were significantly different (Fig. 2B–E; *L. procerum*, β = 0.374; *H. pinicola*, β = 0.697, *F*_1, 74_ = 32.94, *P* < 0.0001; *L. truncatum*, β = 0.586, *F*_1, 71_ = 6.81, *P* = 0.0111; *L. sinoprocerum*, β = 0.446, *F*_1, 72_ = 5.14, *P* = 0.0264).

### Adverse effects of elevated naringenin on the invasive beetle-fungus complex

**Figure 3:**
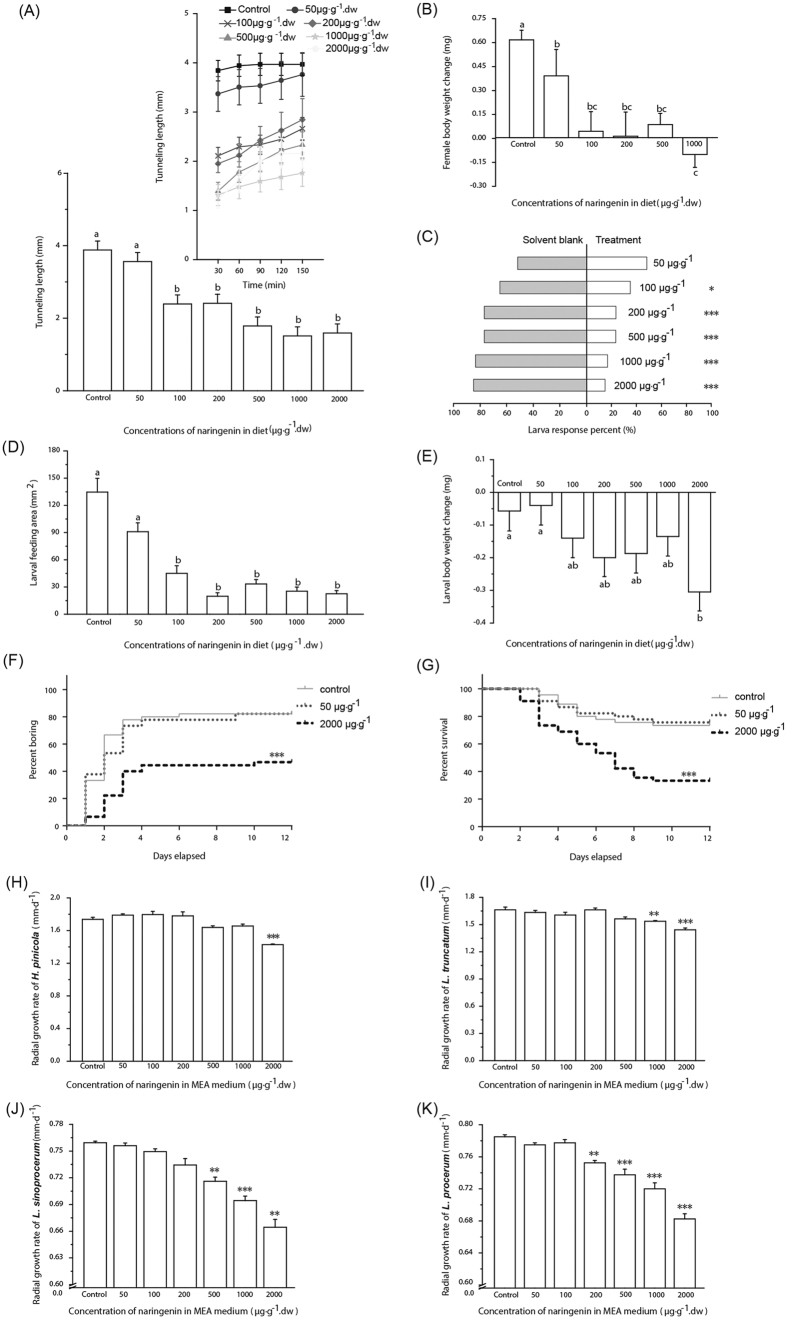
Elevated naringenin concentrations inhibit the beetle-fungus complex. (**A,B**) Mean tunnelling length (+SEM, *n* = 18) and body weight change (+SEM, *n* = 26–30) of female RTB in relation to naringenin. Inset of (**A**) Mean tunnelling length (±SEM) over time. (**C**) Choice of RTB larvae (*n* = 60) for diet with only solvent or with naringenin. (**D,E**) Mean feeding area (+SEM, *n* = 22) and body weight change (+SEM, *n* = 18–20) of RTB larvae expose to naringenin. (**F,G**) Boring and survival rates of RTB larvae (*n* = 45) in the presence of naringenin. (**H–K**) Mean radial growth rates (+SEM, *n* = 4–6) of Chinese-resident fungi and *L. procerum* in the presence of naringenin. In (**A,B**,**D,E**), different letters indicate significant differences between treatments (*P* < 0.05). In (**C**,**F–K**), asterisks indicate significant differences between controls and treatments (*P < 0.05, **P < 0.01, ***P < 0.001).

Female beetle tunnelling length was significantly reduced with elevated naringenin concentrations over 150 min (Fig. 3A; linear mixed-effects model test; treatment, *F*_6, 118.520_ = 14.364, *P* < 0.0001; time, *F*_1, 156.073_ = 48.084, *P* < 0.0001). Female body weight was significantly decreased with elevated naringenin concentrations after 6 h (Fig. 3B; one-way ANOVA, *F*_5, 166_ = 10.143, *P* < 0.0001). Larval beetles were significantly repelled from diet plugs with elevated naringenin concentrations after 6 h (Fig. 3C; 50 μg·g^−1^, 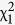 = 0.067, *P* = 0.796; 100 μg·g^−1^, 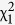 = 5.400, *P* = 0.020; 200 μg·g^−1^, 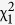 = 17.067, *P* < 0.0001; 500 μg·g^−1^, 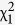 = 17.067, *P* < 0.0001; 1000 μg·g^−1^, 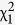 = 26.667, *P* < 0.0001; 2000 μg·g^−1^, 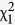 = 29.400, *P* < 0.0001). Larval feeding area (Fig. 3D; Kruskal-Wallis test, 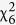 = 84.586, *P* < 0.0001) and body weight (Fig. 3E; two-way ANOVA; treatment, *F*_6, 124_ = 2.346, *P* = 0.035; block, *F*_3, 124_ = 6.559, *P* < 0.001) were significantly decreased with elevated naringenin concentrations after 6 h. Larval boring rate (Fig. 3F; Mantel-Cox test, 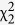 = 21.13, *P* < 0.0001) and survival rate (Fig. 3G; Mantel-Cox test, 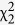 = 23.58, *P* < 0.0001) were significantly decreased with the elevated naringenin concentrations over 12 d.

The radial growth rate of each of the four fungal associates was significantly decreased with elevated naringenin concentrations (Fig. 3H–K; *H. pinicola*, one-way ANOVA, *F*_6, 35_ = 21.112, *P* < 0.0001; *L. truncatum*, one-way ANOVA, *F*_6, 35_ = 12.224, *P* < 0.0001; *L. sinoprocerum*, Brown-Forsythe’s one-way ANOVA, *F*_6, 19.628_ = 44.363, *P* < 0.0001; *L. procerum*, one-way ANOVA, *F*_6, 21_ = 51.393, *P* < 0.0001).

### Naringenin biodegradation by cryptic microbiota with enhanced activity in pinitol, the main soluble carbohydrate of *P. tabuliformis*

**Figure 4:**
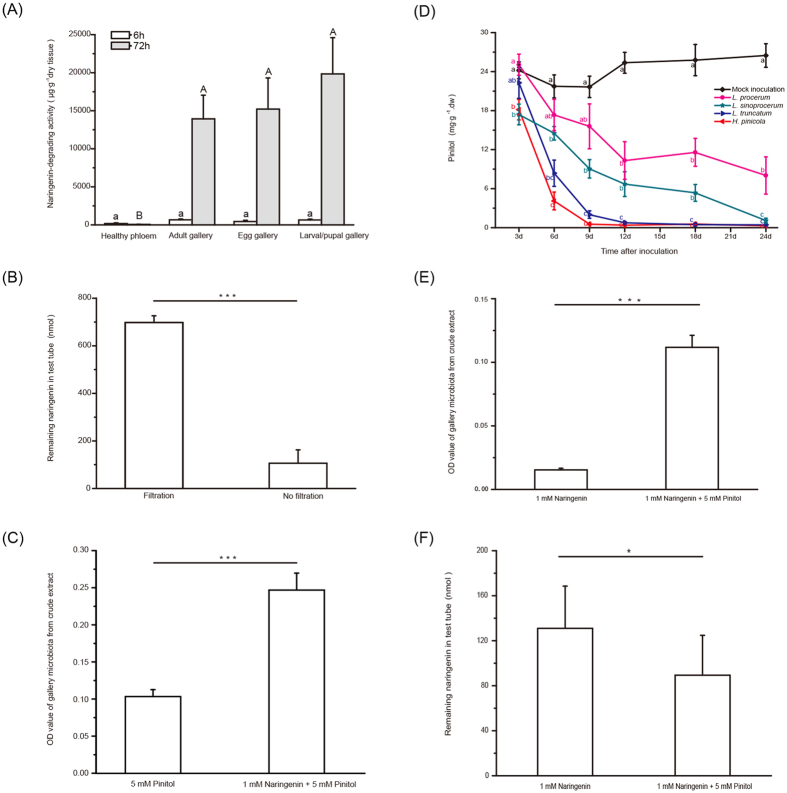
Gallery microbiota degrades naringenin. (**A**) Mean naringenin degradation activities (+SEM, replicates see Experiment 7 in Methods) of healthy phloem and galleries. (**B**) Mean quantities of naringenin (+SEM, *n* = 17) remaining after treatment with gallery crude extract with or without filtration. (**C**) Mean OD_600_ values (+SEM, *n* = 20) of gallery microbiota with or without naringenin in the presence of pinitol. (**D**) Mean concentrations of pinitol (±SEM, *n* = 7–10) in 4-5-yr-old *P. tabuliformis* seedlings inoculated with the three Chinese-resident fungi and the *L. procerum* after 3, 6, 9, 12, 18, and 24 d. (**E,F**) Mean OD_600_ values of gallery microbiota and quantities of naringenin (+SEM, *n* = 18) remaining after treatment with gallery microbiota with or without pinitol in the presence of naringenin. In (**A,D**), different letters indicate significant differences between treatments (*P* < 0.05). In (**B,C,E,F**), asterisks indicate significant differences between two treatments (*P < 0.05, ***P < 0.001).

Crude extracts from *D. valens* galleries caused significantly more naringenin biodegradation than those from healthy phloem after 72 h, but these differences were not significant after just 6 h (Fig. 4A; Kruskal-Wallis tests, 6 h, 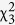 = 7.079, *P* = 0.069; 72 h, 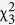 = 23.184, *P* < 0.0001). Filtration through 0.22 μm filters significantly reduced naringenin biodegradation after 72 h (Fig. 4B; paired *t* test; *t*_16_ = 10.292, *P* < 0.001), indicating that naringenin biodegradation of *D. valens* gallery is conferred by cryptic microbiota. OD_600_ values and dry weights of gallery microbiotas with naringenin were significantly higher than those without naringenin (Fig. 4C; paired *t* test; *t*_19_ = −6.68, *P* < 0.001; Supplementary Fig. S8A; paired *t* test; *t*_19_ = −5.45, *P* < 0.001) when grown with pinitol, the main soluble carbohydrate in the phloem of *P. tabuliformis* (Supplementary Fig. S9).

There were significant differences of pinitol concentrations in the phloem of *P. tabuliformis* seedlings 3, 6, 9, 12, 18, and 24 d after inoculation (Fig. 4D; two-way ANOVA, isolate, *F*_4, 234_ = 144.864, *P* < 0.0001; time, *F*_5, 234_ = 51.939, *P* < 0.0001; isolate × time, *F*_20, 234_ = 6.328, *P* < 0.0001). *L. procerum* retained higher concentrations of pinitol than the three Chinese-resident fungi 24 d after inoculation (Fig. 4D; Supplementary Fig. S10). OD_600_ values and dry weights of gallery microbiotas with pinitol were significantly higher than those without pinitol (Fig. 4E; paired *t* test, *t*_17_ = −9.58, *P* < 0.001; Supplementary Fig. S8B; paired *t* test, *t*_17_ = −4.96, *P* < 0.001) when grown with naringenin. Gallery microbiotas with pinitol caused significant naringenin reduction compared to those without pinitol after 72 h (Fig. 4F; Wilcoxon signed ranks test, *Z*_18_ = −2.24, *P* = 0.025).

Fourteen bacterial and 8 yeast species were isolated from gallery microbiotas of 269 galleries (Supplementary Tables S3 and S4), within which 3 bacterial (Supplementary Fig. S11; Brown-Forsythe one-way ANOVA, *F*_14, 33.939_ = 1518.565, *P* < 0.0001) and 6 yeast species (Supplementary Fig. S12; Brown-Forsythe one-way ANOVA, *F*_8, 4.597_ = 1257.845, *P* < 0.0001) caused significant naringenin reductions. Among the 22 microbial species, 5 bacterial and 5 yeast species with pinitol caused significant naringenin reduction compared to the strain without pinitol after 72 h (Supplementary Fig. S13).

## Discussion

**Figure 5:**
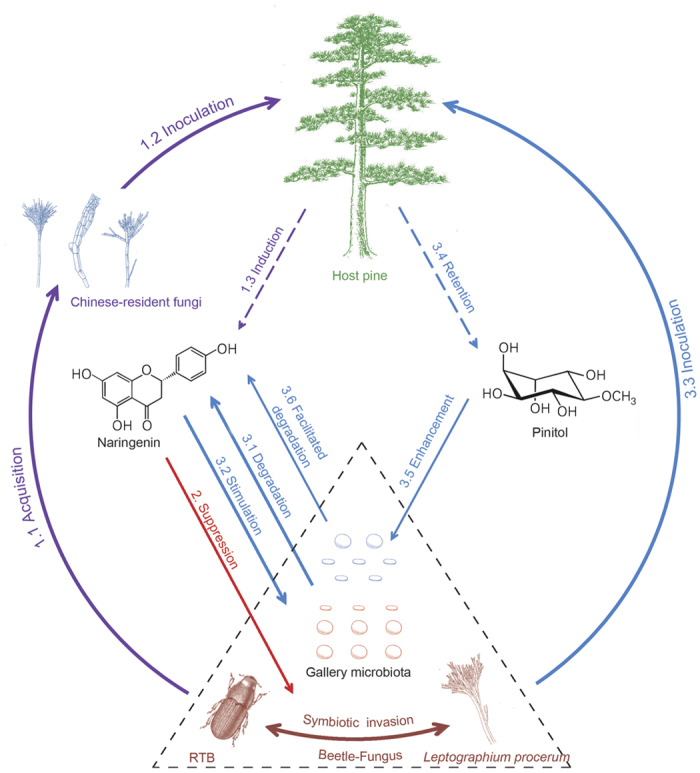
Complex interactions mediated by phytochemicals among host pine, red turpentine beetle, ophiostomatoid fungi and associated bacteria and yeasts. 1.1–1.3 (purple lines): Chinese-resident fungi acquired by RTB in China are inoculated to specifically induce naringenin in the phloem of host pines. 2 (red line): Naringenin suppresses the invasive RTB-*L. procerum* complex (in brown). 3.1–3.2 (blue lines): RTB gallery microbiota degrades naringenin, and naringenin stimulates the growth of gallery microbiota. 3.3–3.4 (blue lines): *L. procerum* is inoculated to induce retention of pinitol. 3.5–3.6 (blue lines): Pinitol enhances the growth and facilitates naringenin biodegradation of gallery microbiota. Dashed triangle (black line): A close tripartite symbiosis of RTB, *L. procerum* and gallery microbiota (Supplementary Fig. S14 and Table S6) enhances the invasion potential of the beetle-fungus complex in China.

Recent studies have demonstrated various essential roles of microbial symbionts for invasive pests in colonizing new host plants^7^,^38^,^39^,^40^, yet the dynamic interactions between invasive pest, microbial symbionts and new host plant appear to be more complicated than previously thought. In our system, we present evidence that the invasive RTB-*L. procerum* symbiotic complex appears to be challenged by host pine inducible phenolic resistance but this resistance can be mitigated by cryptic microbiotas in the beetle galleries. *H. pinicola*, *L. truncatum* and *L. sinoprocerum*, species responsible for the induced phenolic resistance, have not been reported as the fungal associates of RTB in the US, whereas seem to be unfortunately acquired by Chinese RTB populations^41^,^42^,^43^ (Fig. 5, lines 1.1–1.3; Supplementary Table S1). The ecological roles of flavonoids have been paid less attention than primarily studied stilbenes in bark beetle interactions; here, the increased naringenin present in host pines is shown to suppress the performance of both RTB and the fungal associate *L. procerum* (Fig. 5, line 2). This induced defensive flavonoid then, however, would be degraded by RTB gallery microbiota (Fig. 5, line 3.1). RTB populations might have tended to loosely associate with those resident fungi during invasion, considering that the invasive fungus *L. procerum* presents more cooperative characteristics with gallery microbiota than do the resident fungi (Fig. 5, lines 3.2–3.6). The association between naringenin-biodegrading microbiota and RTB-*L. procerum* symbiotic complex is quite close in the field (Supplementary Fig. S14, Tables S5 and S6), which implies the prevalence of naringenin-biodegrading microbiota that provides increased ability for this beetle-fungus complex to overcome host plant resistance (Fig. 5). Our study is the first step toward investigating possible mechanisms for reduced biotic resistance to symbiotic invasions, which could be a major contributing factor in the naturalization of an invasive pest system in the introduced ranges.

Caution must be taken when making inferences on the ecological interactions in native pine forests, since these experiments were conducted with pine seedlings and beetle/symbionts bioassays *in vitro*. Although it was evident that naringenin concentrations were positively correlated to pine seedling stem diameters (or ages) (Fig. 2B–E), it is still unknown whether differential inductions of naringenin amongst fungi in *P. tabuliformis* seedlings can be extrapolated to those in mature pines; further, the potential combinatorial effects of these fungi that may synergize or antagonize naringenin induction remains unclear. In spite of good agreement between *in vitro* and *in vivo* experiments as shown in other bark beetle/fungus cases of phenolic resistances^22^,^44^,^45^, observations are needed on the performance of this beetle-fungus complex under elevated naringenin in mature *P. tabuliformis* in the future. Furthermore, this putative reduced biotic resistance should be linked to the time lag among introductions of RTB-*L. procerum* into new regions in China, its acquisition of resident fungal associates, its population dynamics and the compositional change of the cryptic microbiota in the field at larger temporal and spatial scales.

Microbial mediation of pine resistance may be more complicated than the results presented. Fungal infection of *P. tabuliformis* could reshuffle the biosynthesis of primary and secondary metabolites in the phloem. For example, in our results, the trade-off in biogenesis of pinitol (which is a cyclic polyol) and naringenin after the invasive/resident fungal infections potentially evidenced a connective pathway from cyclitols to phenolics in *P. tabuliformis*, which may be seen from another cyclitol, shikimic acid, that has been inferred in relation to production of many phenolics including naringenin in other systems^46^,^47^,^48^. The ecological role of gallery microbiota is rather intricate, especially when integrating terpenoid compounds into this work. Previous studies indicated the monoterpene 3-carene^35^,^36^ and diterpene resin acids^37^ appear to facilitate invasion success by the beetle-fungus complex; however, certain genera, e.g. *Serratia* and *Rahnella* in gallery microbiota of RTB were reported to metabolize monoterpenes and diterpene acids in another bark beetle system^30^. Compared to genera such as *Burkholderia* and *Novosphingobium* that were highly efficient in naringenin biodegradation (Supplementary Fig. S11), *Serratia* and *Rahnella* may specialize in terpenoid metabolism. Under this circumstance, it seems that gallery microbiota plays dual or multiple roles in processing pine defensive compounds, which is hard to absolutely define as “good” or “bad”. Given that terpenoids and phenolics mix in oleoresin, influence of terpenoids on microbial capacity in naringenin biodegradation needs to be evaluated in the future.

The discovery of identity and function of naringenin-biodegrading gallery microbiota went through three stages. First, utilizing RTB galleries collected from naturally infested forests, we found gallery tissues that exhibited higher naringenin-biodegrading activities after 72 h, compared to tissues from healthy phloem (Fig. 4A). Then, crude extracts from gallery tissues were filtered through 0.22 μm filters, which largely lost their capacity to degrade naringenin (Fig. 4B), revealing that naringenin degradation could be explained by microbial activities. Thus, we applied isolation method using naringenin as sole carbon source, an enrichment process for picking out naringenin-tolerant and naringenin-biodegrading species (Supplementary Table S5). Naringenin is commonly known to be antimicrobial^49^,^50^, whereas growth of the microbiota increased in response to this compound, possibly due to those microbes existing in RTB galleries (Fig. 4C). Bacteria and yeasts have been considered as novel partners in pine-bark beetle-ophiostomatoid fungi interactions^25^,^51^ and were also reported in RTB associated environments^52^,^53^,^54^. Many ecological functions of them have been disclosed; however, phenolic degrading microbes seem “cryptic” and their roles were seldom emphasized in bark beetle systems. For example, in this study, compared to naringenin-tolerant genera (e.g. *Serratia*, *Pantoea* and *Raoultella* etc.) that have often been found associated with RTB or other bark beetles, genera of microbiota capable of naringenin biodegradation (such as *Burkholderia*, *Novosphingobium* and yeast genus *Rhodotorula*) were previously regarded as minor ones. In another work, using community pyrosequencing, we found the frequency and abundance of *Burkholderia* and *Novosphingobium* were nearly equivalent to the more commonly seen genera (unpublished data). These “cryptic” genera may be of more importance in microbial ecology of RTB or even other bark beetles than previously thought.

Although naringenin-enrichment methods for microbial isolation helped us focus on particular microbes that potentially degrade naringenin, we did not always obtain highly effective species from the galleries that showed high biodegrading activities (Supplementary Table S6). This implied that traditional methods might overlook important microbes during the manual isolation process or that the production of high degrading activity in gallery microbiota might be more complicated than the actions of individual microbial species. The exact genes or metabolic pathways responsible for phenolics or even specifically for naringenin biodegradation that were operated by combinations of those microbes need to be studied in the future by metagenomics, an approach successfully applied in other bark beetle or insect systems to uncover the composition and functioning of their symbiotic microbiotas^55^,^56^.

The naringenin-biodegrading gallery microbiota may come partly from the RTB gut, since bacterial species of the genera *Rahnella*, *Pseudomonas* and *Burkholderia* as well as the yeast species *Cyberlindnera americana* and *Kuraishia molischiana* are frequently found in gut tissues from RTB and other bark beetle species^26^,^52^,^53^,^55^,^56^,^57^,^58^. The high naringenin-degrading *Novosphingobium* and *Burkholderia* have also been reported in oral secretions of bark beetles^59^, suggesting another possible source of the microbiota. *Burkholderia* and *Pseudomonas* found in the microbiota may also be vectored by RTB from associated environments, considering the presence of these genera in the pine rhizosphere^60^,^61^ and the behaviour of RTB to colonize and develop in the roots in addition to the main stem of pines^34^.

In general, despite the fact that there need more evidence to illustrate the mechanisms underlying reduced biotic resistance, our results presented a possible scenery that microbial symbionts functioned in such a diverse way for invasive insect pest through mediations of host plant resistance. Besides, this work involved an intricate cross-kingdom interactions (plant-insect-fungi-bacteria) showing more fascinating “warfare” between plant and herbivore than previously realized. Our findings also suggested that the full range of functions of microbes associated with invasive species should be considered in assessing the mechanisms underlying symbiotic invasion success.

## Methods

### Seedlings, fungal associates, and beetles

*P. tabuliformis* seedlings (diameter of 1-2-yr-old seedlings: Mean ± S.E = 5.87 ± 0.06 mm; diameter of 2-3-yr-old seedlings: Mean ± S.E = 7.08 ± 0.05 mm; diameter of 3-4-yr-old seedlings: Mean ± S.E = 8.09 ± 0.07 mm; diameter of 4-5-yr-old seedlings: Mean ± S.E = 9.99 ± 0.12 mm; measured at 2cm above the soil line) were cultivated in plastic pots (diameter: 12 cm) and transferred to a glasshouse (air temperature: 25 °C; relative humidity: 60%; 12 h photoperiod) for at least one month before the inoculation experiments.

The 11 fungal associates of *D. valens* used in this study were previously isolated from beetles or beetle galleries in infested *P. tabuliformis* forests in China and cultures were collected in the Forestry and Agricultural Biotechnology Institute, University of Pretoria and the Belgian Coordinated Collections of Microorganisms^41^,^42^. These fungal species (*L. procerum* from Chinese-invasive group; *Ophiostoma floccosum*, *L. koreanum*, *O. abietinum* and *O. ips* from shared group; *O. rectangulosporium*, *O. minus*, *L. pini-densiflorae*, *L. sinoprocerum*, *L. truncatum* and *Hyalorhinocladiella pinicola* from Chinese-resident group; Supplementary Table S1) accounted for 86.7% of total field isolations^41^,^42^,^43^.

Female *D. valens* beetles were collected from traps baited with *D. valens* kairomone lure (+)-(3)-carene in a naturally attacked forest at Tunlanchuan Forest Station (37°48′N, 111°44′E; average elevation 1400 m; *P. tabuliformis* trees: 3278 ha), Shanxi province. Second to third instar *D. valens* larvae were randomly collected from the beetle galleries of infested host pines at Tunlanchuan Forest Station.

### Seedling inoculation and quantification of phenolic compounds

It is both ecologically dangerous and illegal (because of the National Natural Forest Protection Project in China) to operate inoculation experiments with invasive beetle and fungi or even artificial damages in uncontrolled, mature *P. tabuliformis* forests. Therefore, we chose to use *P. tabuliformis* seedlings in controlled conditions as a safe, legal alternative to large scale inoculations in natural forests. We performed single inoculation on one seedling in order to avoid excessive mechanical wound to the stem^35^,^36^,^62^,^63^. Although some studies showed ontogenetic differences in resistances between seedlings and mature plants^64^,^65^, other reports indicated that necrotic lesions from seedlings by several ophiostomatoid fungi are in agreement with those from mass-inoculated mature trees^62^,^63^. The tree size/fungal dosage ratio applied to mass-inoculated mature trees was found similar with that used in our *P. tabuliformis* seedlings (for example^66^, 20-yr-old unpruned *P. sylvestris* with 170 inoculations, the tree size/fungal inoculation ratio is 352, while the ratio we used as 4-5-yr-old seedling/a 5 mm diameter plug is 440).

To test the effect of fungi on pine phenolic defences and to select Chinese-resident fungi for subsequent experiments, inoculation assays were carried out using the eleven fungal associates of *D. valens* in China on 4-5-yr-old seedlings in May 2011. Seedlings received single inoculations with one of the fungal species or mock inoculation (*n* = 3 to 5 per treatment) applied by using a plug of 2% MEA alone (without fungi) on seedlings to serve as control. Infectious phloem materials (between 5 mm above and below inoculation point) were sampled at 21 d after inoculation (Experiment 1).

To test the effect of fungi on pine phenolic defences across time, in July 2011, we performed inoculation assays using Chinese-invasive fungus *L. procerum* and three Chinese-resident fungi (*H. pinicola*, *L. truncatum* and *L. sinoprocerum*) on 4-5-yr-old seedlings. The three Chinese-resident fungi accounted for 63.1% of field isolations of Chinese-resident group^41^,^42^,^43^. Seedlings received single inoculations with one of the four fungal species or mock inoculation (*n* = 7–10 per treatment in each time point). Infectious phloem materials were obtained at 3, 6, 9, 12, 18 and 24 d after inoculation (Experiment 2).

To test the effect of tree size on accumulation of naringenin, seedlings with ascending diameters across pine ages (1-2- to 4-5-yr-old seedlings) received single inoculations with one of the four fungal species (*n* = 8–10 per treatment in each pine age). Infectious phloem tissues were obtained at 24 d after inoculation (Experiment 3).

Before extraction, the phloem tissue was ground to powder in liquid N and washed with pentane (Sigma) to remove the resinous compounds. Phenolic compounds (two monoaryls *p*-coumaric acid and ferulic acid, two flavonoids taxifolin and naringenin and three stilbenes resveratrol, pinosylvin and pinosylvin monomethyl ether) were extracted from HPLC-grade methanol (JK Chemical Corporation, Beijing) and analyzed using HPLC (Agilent 1100, USA) as previously described^67^ with detailed procedures presented in Supplementary Methods Experiment 1. The phenolic polymer lignin was detected by spectrophotometry (Beckman Coulter, USA) and quantified by standard calibration curves with five different concentrations of lignin (TCI, Japan) according to the methods described^68^.

### Bioassays on beetle and fungal associates under increased naringenin *in vitro*

In order to measure the effects of elevated levels of naringenin on beetle and fungal performances, we performed experiments on phloem media (for beetles) and MEA (for fungi) amended with naringenin to yield concentrations 0, 50, 100, 200, 500, 1000 and 2000 μg·g^−1^ of media dry weight, thus covering the concentration gradient induced by fungal associates from seedlings (Fig. 2).

To test tunnelling length of female *D. valens* under elevated levels of naringenin, phloem medium was prepared as described^43^ and then molten media amended with naringenin (dissolved in ethyl acetate) were transferred into a transparent glass tube (4 mm inner diameter, 50 mm in length) with enough space left at the open end for one beetle. Beetles were surface-sterilized and inserted head-down into each tube (*n* = 18 per treatment) then sealed with parafilm^22^. Tunnelling lengths were measured at half-hour intervals and longer tunnels indicate greater acceptance of substrate. To test body weight change of females, initial body weight of each beetle was recorded before insertion into tube containing naringenin amended phloem medium (*n* = 26–30 per treatment) and the ultimate body weight was recorded after 6 h (Experiment 4).

To test diet choice of *D. valens* larvae under elevated levels of naringenin, we placed one diet plug (diameter: 1.5 cm) with only solvent and the other with naringenin in a 35 mm Petri dish (two-choice bioassay). One sterilized larva was placed equidistant from the two plugs^69^. Petri dishes (*n* = 60 per treatment) were kept in 25 °C in darkness. Larvae were allowed to choose diet plugs and results were recorded after 6 h. In no-choice bioassays, to test body weight change of larvae (*n* = 18–20 per treatment), larvae were grouped into four blocks according to their initial weight, randomized into amended phloem media in respective blocks as one larva per Petri dish and reweighed after 6 h; to test feeding areas (*n* = 22 per treatment), larvae were removed from amended phloem media after 6 h and the feeding areas were photographed and measured using software Image J. It has been found that conifer phenolics have a clear antifeedant effect that acts quickly on bark beetle^22^, so we set experimental time of many assays as short as possible (within 6 hours); and we further tested the antifeedant effect of naringenin in longer time: to test long-term boring rate and survival (*n* = 45 per treatment), larval performances on phloem media amended with solvent (control) and two levels of naringenin (50 μg·g^−1^ and 2000 μg·g^−1^ of diet dry weight) were recorded every two days (Experiment 5).

To test radial growth rates of the four fungal associates under elevated levels of naringenin, we first dissolved naringenin in ethyl acetate and then mixed it into autoclaved 2% MEA to yield the appropriate naringenin concentrations. Ethyl acetate alone was used as control. Five mm fungal plugs were taken from the margin of actively growing fungal cultures and then placed on the centre of 90 mm diameter plates of MEA (*n* = 4–6 per treatment), ensuring that aerial mycelia were in contact with media^36^. Plates were incubated in the dark at 25 °C and growth was measured daily (Experiment 6).

### Naringenin biodegradation

To test naringenin biodegradation by cryptic microbiotas in beetle galleries, we collected 111 *D. valens* galleries including 64 adult galleries, 18 egg galleries, 27 larval galleries, and 2 pupal galleries, from infested trees of a natural *P. tabuliformis* forest at Tunlanchuan Forest Station in July 2012. Samples were stored in sterile Eppendorf tubes at 4 °C and immediately transferred for experiments. Crude extracts (500 μl) containing putative microbiotas were obtained from one portion of the 111 gallery tissues and transferred to 1 ml of inorganic culture solution containing 1 mM naringenin as sole carbon source, methods referred to previously described^70^. Healthy phloem discs were also collected from 12 uninfested mature pines to see if putative innate phloem endophytes are responsible for naringenin biodegradation as well. After 6 h and 72 h of incubation, naringenin degraded by crude extracts of *D. valens* galleries and healthy phloem was detected and quantified by HPLC. To further determine that naringenin biodegrading activity is conferred by microbial symbionts distributed in galleries, crude extracts from 17 galleries were divided into one part with filtration through sterile 0.22 μm (pore size) filters and another part with no filtration. After 72 h of incubation, remaining naringenin was quantified by HPLC. Pinitol is a main soluble carbohydrate in the phloem of *P. tabuliformis* seedlings (Supplementary Fig. S9) and also in mature stems^54^. To test the effect of naringenin on the growth of gallery microbiota, crude extracts (40 μl) from 20 galleries were added in pinitol solutions (5 mM) with and without naringenin (1 mM). After 72 h of incubation at 30 °C, microbial solutions were transferred from glass culture tubes to 96 well flat-bottom microtiter plates to determine the final concentrations by measuring optical density at 600 nm (OD_600_) with a microplate reader (SpectraMax Plus, USA) and dry weights of the microbes were measured to the nearest microgram (μg) using an analytical balance (Mettler-Toledo, Switzerland) (Experiment 7).

To test the effect of the four fungi on pinitol retention in pine seedlings across time, we performed inoculation assays on 4-5-yr-old pine seedlings using the four fungal species (*n* = 7–10 per treatment in each time point) following procedures as described above, in August 2012. Pinitol was extracted from phloem tissues by methanol containing ribitol as internal quantitative standard, heated at 70 °C and separated by polar phase fractionation, followed by derivatization. Derivatized carbohydrates were identified and quantified by GC-MS and GC-FID (Agilent 7890A, USA) as described previously^71^ (Experiment 8).

To test the effect of pinitol on naringenin biodegradation of gallery microbiota, crude extracts (40 μl) from 18 galleries were added in naringenin solutions (1 mM) with and without pinitol (5 mM). After 72 h of incubation at 30 °C, final concentrations and dry weights of microbes were determined by measuring optical density at 600 nm and by using analytical balance respectively, and the remaining naringenin was quantified by HPLC (Experiment 9).

Detailed procedures for corresponding experiments mentioned above were presented in Supplementary Methods and additional information was placed into Supplementary Extended Methods.

### Data analysis

Differences in two treatments were determined using *t* tests (Experiments 7 and 9). Differences of more than two groups of cases were analyzed by ANOVA (Experiments 1, 2, 4–8). For all *t* tests and ANOVA analyses, we tested the normal distribution (Kolomogorov-Simirnov test) and homogeneity (Levene’s test) of the variances of the responses for each treatment. Bonferroni *post hoc* tests were used for pair-wise comparisons following ANOVAs (Experiments 4 and 6). Datasets that did not conform to normality or had unequal variance were rank-transformed for one-way ANOVA (Experiment 4), one-way Brown-Forsythe’s ANOVA tested (Experiment 6), or non-parametric Kruskal-Wallis tested (Experiments 1, 2, 5, 7 and 8), with Student-Newman-Keuls *post-hoc* test, Dunnett’s T3 tests, or Mann-Whitney *U* tests using Bonferroni correction, respectively. The relationships between naringenin concentration and seedling diameter were examined by simple linear regression analysis (Experiment 3). Comparisons of regression coefficients between each of Chinese-resident fungi and the Chinese-invasive *L. procerum* were conducted by analysis of covariance. A significant interaction term between fungal associate inoculation and diameter indicated that the slopes of the two relationships differed significantly (Experiment 3). Preference behaviour of *D. valens* (Experiment 5) was tested using a chi-squared test. The survival and boring rate of larvae (Experiment 5) were calculated by Kaplan-Meier survival analysis (GraphPad Prism Software, Inc., San Diego, CA). All tests were carried out by using IBM SPSS 20 (SPSS Inc., Chicago, IL, USA) and SAS (SAS Institute Inc., Cary, NC, USA). Detailed statistics for corresponding experiments were presented in Supplementary Methods.

## Additional Information

**How to cite this article**: Cheng, C. *et al*. Does cryptic microbiota mitigate pine resistance to an invasive beetle-fungus complex? Implications for invasion potential. *Sci. Rep*. **6**, 33110; doi: 10.1038/srep33110 (2016).

## Supplementary Material

Supplementary Information
