# Supplementary material for: Does cryptic microbiota mitigate pine resistance to an invasive beetle-fungus complex? Implications for invasion potential
Source: Sci Rep. 2016 Sep 13;6:33110. doi: 10.1038/srep33110 (PMC5020614; doi:10.1038/srep33110)
Supplement: Supplementary Information [file srep33110-s1.pdf]

Does cryptic microbiota mitigate pine resistance to an invasive beetle-fungus complex? Implications for invasion potential

Chihang Cheng, Letian Xu, Dandan Xu, Qiaozhe Lou, Min Lu*, Jianghua Sun*

Content:

Supplementary Figure S1-S14

Supplementary Table S1-S6

Supplementary Methods

Supplementary Extended Methods

Figures

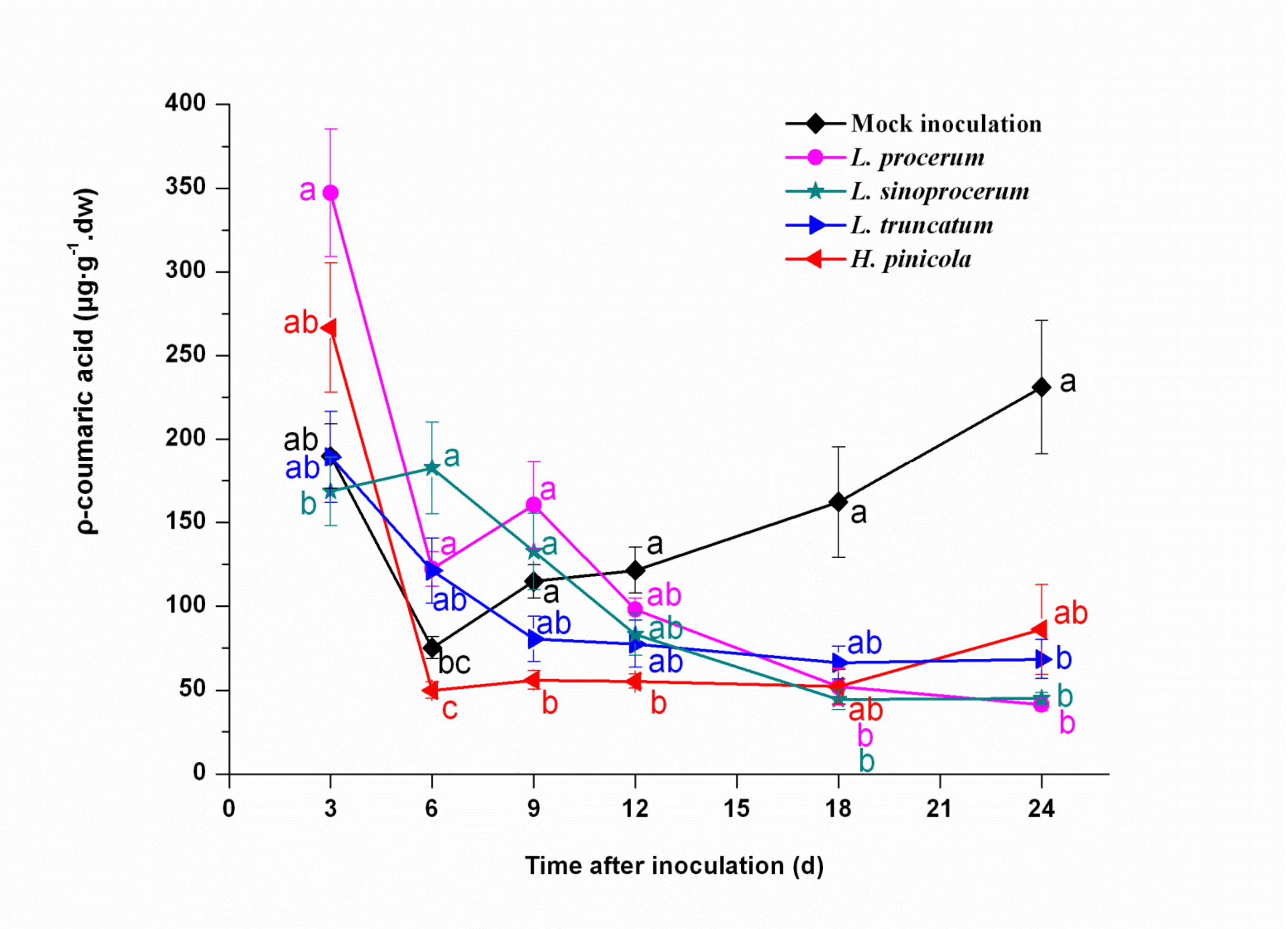

Figure S1 | Mean concentrations of *ρ*-coumaric acid (± SEM, *n* = 7-10) from *P. tabuliformis* seedling phloem inoculated with three Chinese-resident fungi and *L. procerum* associated with RTB. Two-way ANOVA was used to analysis with time and isolate as fixed factors. Type III test of fixed effects results: isolate, $F_{4, 224} = 7.559$, $P < 0.0001$; time, $F_{5, 224} = 38.742$, $P < 0.0001$; isolate × time, $F_{20, 224} = 7.311$, $P < 0.0001$. 3 d: Kruskal-Wallis test, $\chi^2_4 = 14.070$, $P < 0.01$; 6 d: Kruskal-Wallis test, $\chi^2_4 = 26.277$, $P < 0.0001$; 9 d: Kruskal-Wallis test, $\chi^2_4 = 17.728$, $P < 0.01$; 12 d: one-way ANOVA, $F_{4, 34} = 4.902$, $P < 0.01$; 18 d: Kruskal-Wallis test, $\chi^2_4 = 14.790$, $P < 0.01$; 24 d: Kruskal-Wallis test, $\chi^2_4 = 23.182$, $P < 0.001$. Different letters indicate significant differences between isolates ($P < 0.05$).

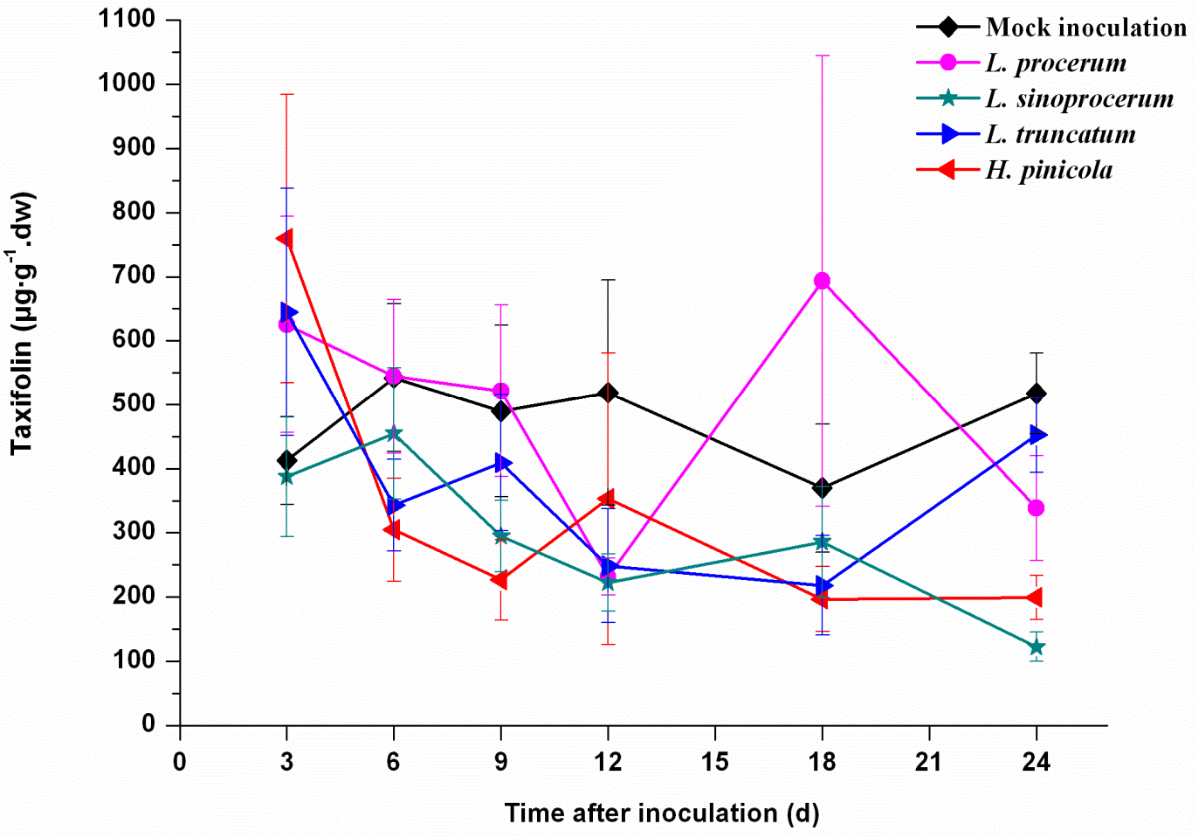

Figure S2 │ Mean concentrations of taxifolin (± SEM, *n* = 7-10) from *P. tabuliformis* seedling phloem inoculated with three Chinese-resident fungi and *L. procerum* associated with RTB. Two-way ANOVA was used to analysis with time and isolate as fixed factors. Type III test of fixed effects results: isolate, $F_{4, 224} = 2.398$, $P = 0.051$; time, $F_{5, 224} = 2.414$, $P = 0.037$; isolate × time, $F_{20, 224} = 1.032$, $P = 0.426$. No differences were found between isolates.

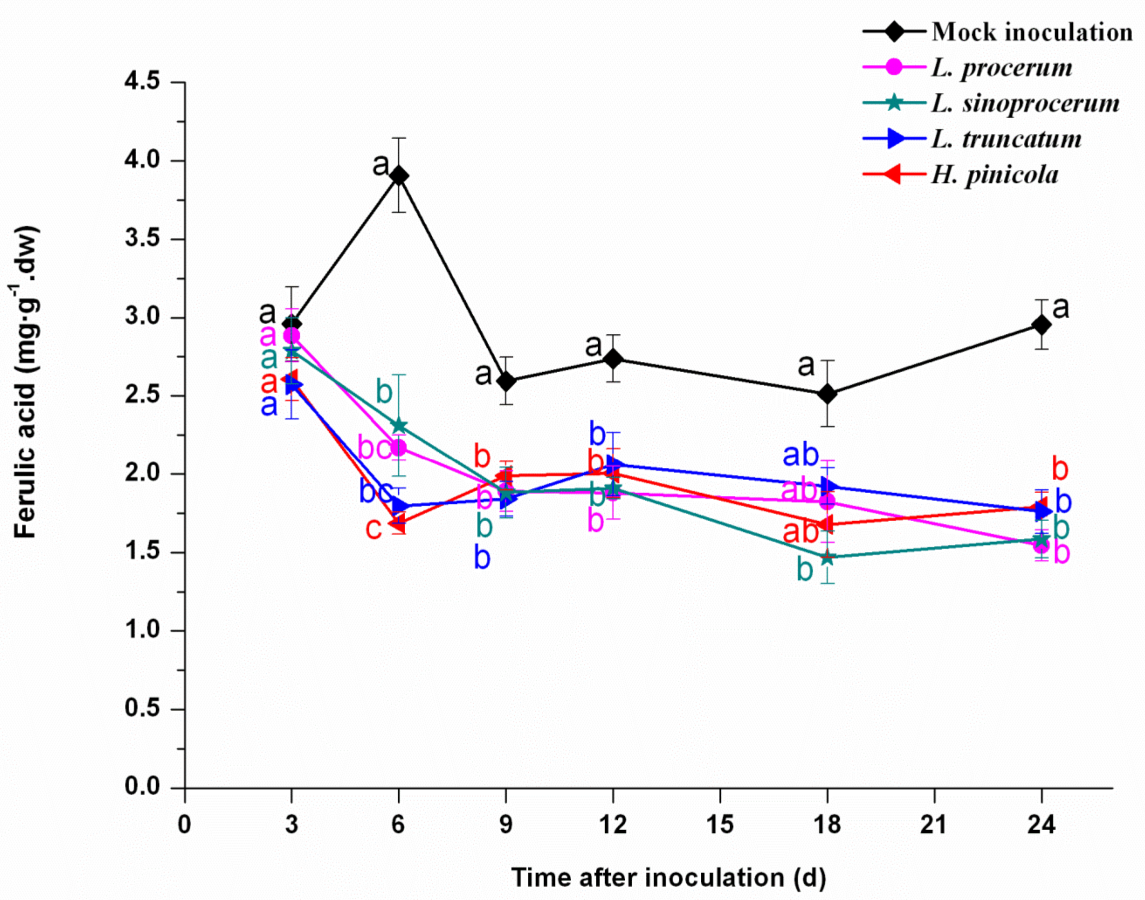

Figure S3 | Mean concentrations of ferulic acid (± SEM, *n* = 7-10) from *P. tabuliformis* seedling phloem inoculated with three Chinese-resident fungi and *L. procerum* associated with RTB. Two-way ANOVA was used to analysis with time and isolate as fixed factors. Type III test of fixed effects results: isolate, $F_{4, 224} = 37.887$, $P < 0.0001$; time, $F_{5, 224} = 18.957$, $P < 0.0001$; isolate × time, $F_{20, 224} = 3.042$, $P < 0.0001$. 3 d: one-way ANOVA, $F_{4, 41} = 0.699$, $P = 0.597$; 6 d: Kruskal-Wallis test, $\chi^2_4 = 23.819$, $P < 0.0001$; 9 d: one-way ANOVA, $F_{4, 41} = 5.878$, $P < 0.001$; 12 d: one-way ANOVA, $F_{4, 34} = 5.913$, $P < 0.01$; 18 d: one-way ANOVA, $F_{4, 38} = 3.837$, $P < 0.05$; 24 d: one-way ANOVA, $F_{4, 34} = 23.157$, $P < 0.0001$. Different letters indicate significant differences between isolates ($P < 0.05$).

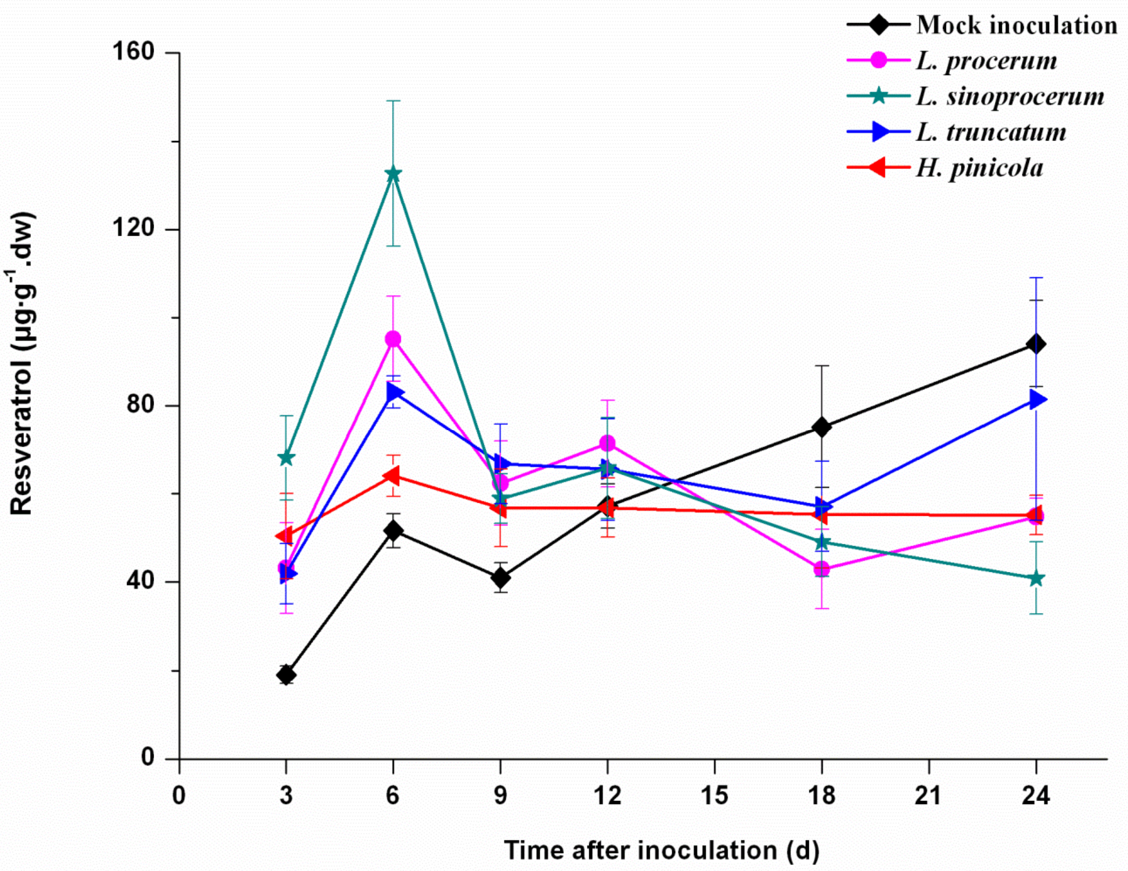

Figure S4│Mean concentrations of resveratrol (± SEM, *n* = 7-10) from *P. tabuliformis* seedling phloem inoculated with three Chinese-resident fungi and *L. procerum* associated with RTB. Two-way ANOVA was used to analysis with time and isolate as fixed factors. Type III test of fixed effects results: isolate, $F_{4, 224} = 2.085$, $P = 0.084$; time, $F_{5, 224} = 9.813$, $P < 0.0001$; isolate × time, $F_{20, 224} = 4.025$, $P < 0.0001$. No differences were found between isolates.

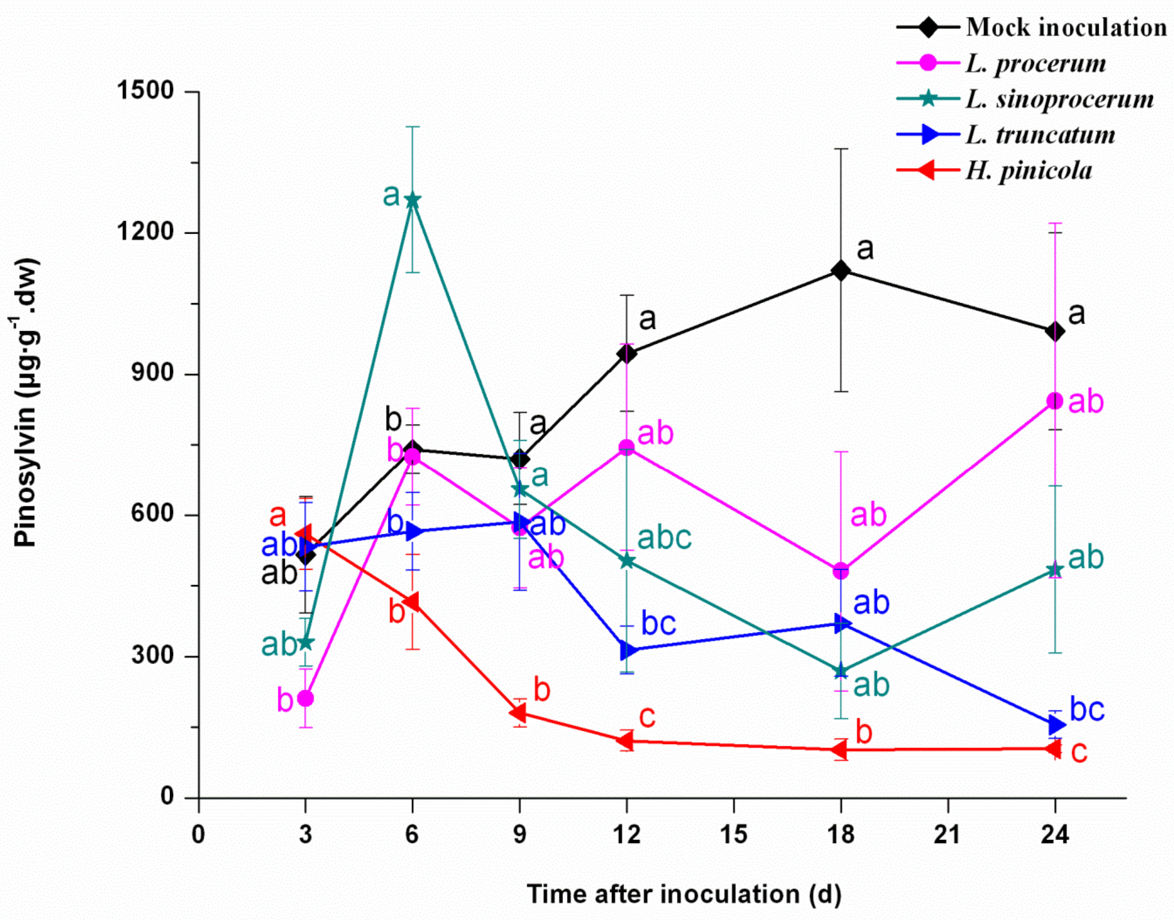

Figure S5 │ Mean concentrations of pinosylvin (± SEM, *n* = 7-10) from *P. tabuliformis* seedling phloem inoculated with three Chinese-resident fungi and *L. procerum* associated with RTB. Two-way ANOVA was used to analysis with time and isolate as fixed factors. Type III test of fixed effects results: isolate, $F_{4, 224} = 13.099$, $P < 0.0001$; time, $F_{5, 224} = 2.670$, $P = 0.023$; isolate × time, $F_{20, 224} = 2.734$, $P < 0.001$. 3 d: one-way ANOVA, $F_{4, 41} = 3.507$, $P < 0.05$; 6 d: one-way ANOVA, $F_{4, 36} = 9.646$, $P < 0.0001$; 9 d: Kruskal-Wallis test, $\chi^2_4 = 16.175$, $P < 0.01$; 12 d: Kruskal-Wallis test, $\chi^2_4 = 20.930$, $P < 0.001$; 18 d: Kruskal-Wallis test, $\chi^2_4 = 15.393$, $P < 0.01$; 24 d: Kruskal-Wallis test, $\chi^2_4 = 22.912$, $P < 0.001$. Different letters indicate significant differences between isolates ($P < 0.05$).

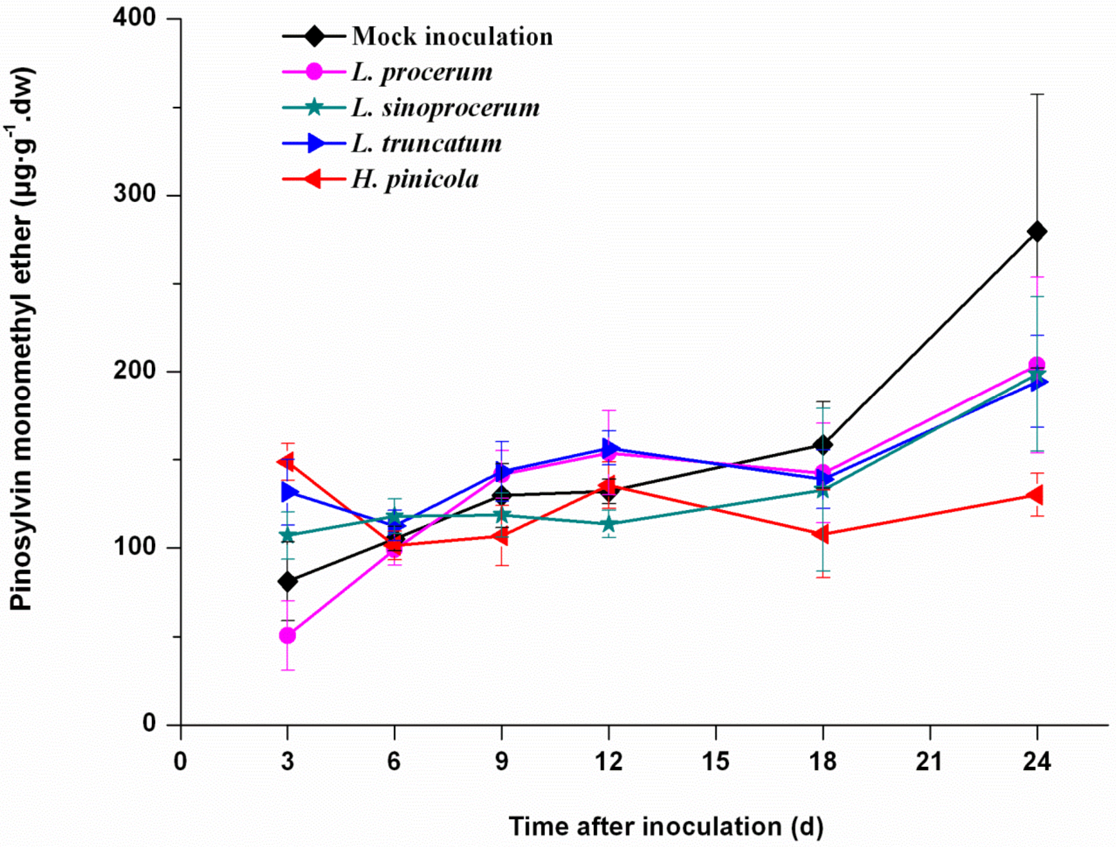

Figure S6 | Mean concentrations of pinosylvin monomethyl ether (± SEM, *n* = 7-10) from *P. tabuliformis* seedling phloem inoculated with three Chinese-resident fungi and *L. procerum* associated with RTB. Two-way ANOVA was used to analysis with time and isolate as fixed factors. Type III test of fixed effects results: isolate, $F_{4,\ 224} = 1.009$, $P = 0.404$; time, $F_{5,\ 224} = 8.492$, $P < 0.0001$; isolate × time, $F_{20,\ 224} = 1.351$, $P = 0.149$. No differences were found between isolates.

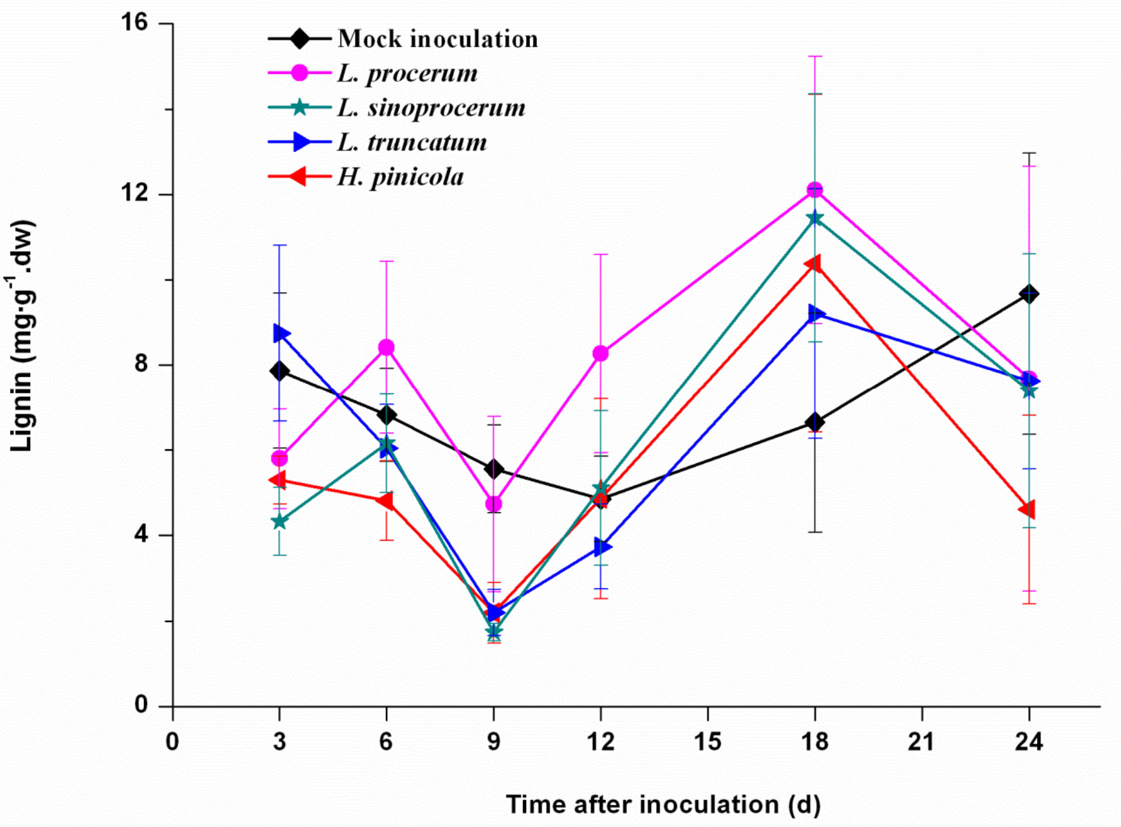

Figure S7 | Mean concentrations of lignin (± SEM, *n* = 7-10) from *P. tabuliformis* seedling phloem inoculated with three Chinese-resident fungi and *L. procerum* associated with RTB. Two-way ANOVA was used to analysis with time and isolate as fixed factors. Type III test of fixed effects results: isolate, $F_{4, 224} = 1.147$, $P = 0.335$; time, $F_{5, 224} = 5.487$, $P < 0.0001$; isolate × time, $F_{20, 224} = 0.633$, $P = 0.886$. No differences were found between isolates.

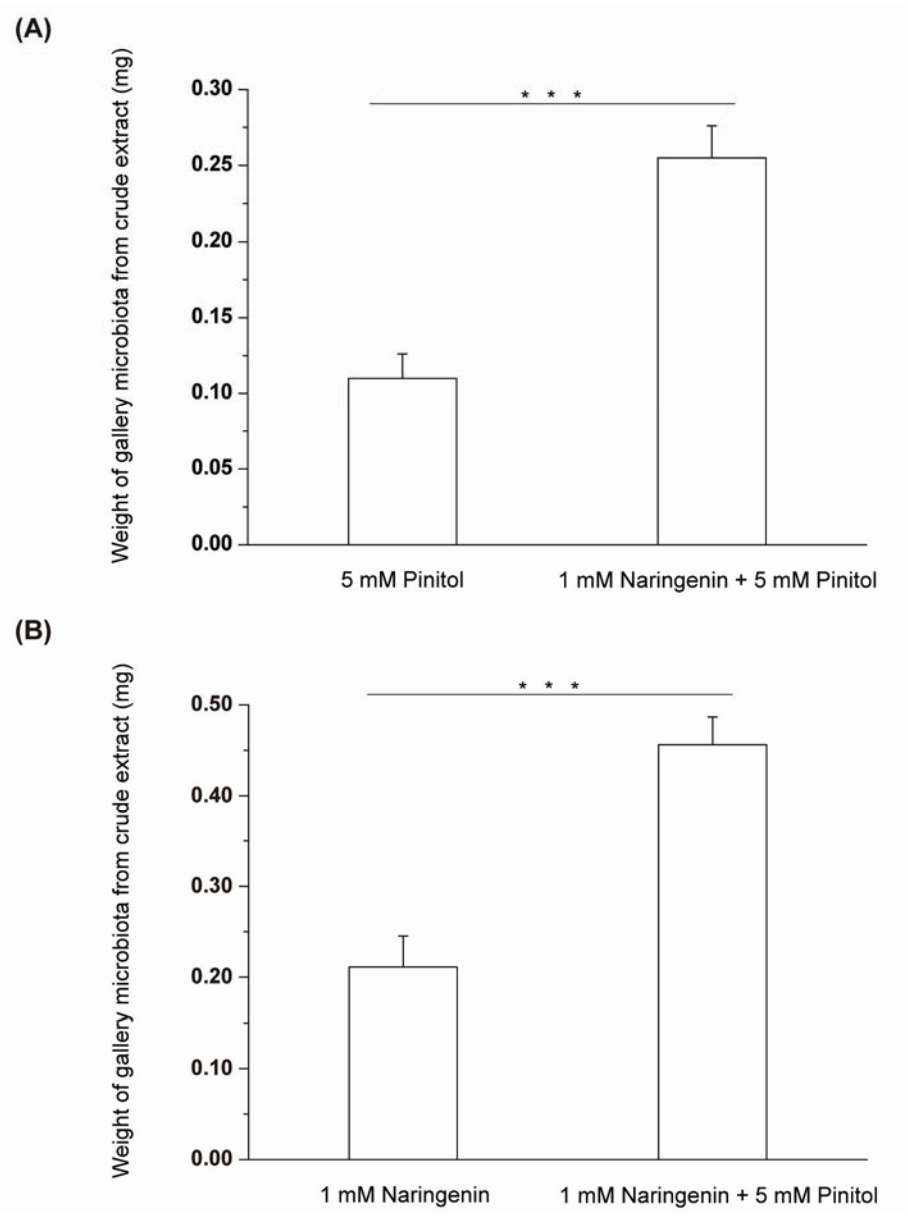

Figure S8 │ The effects of naringenin and pinitol on dry weight changes of gallery microbiotas. (**A**) Mean dry weights (+ SEM, $n = 20$) of gallery microbiota with or without naringenin in the presence of pinitol. (**B**) Mean dry weights (+ SEM, $n = 20$) of gallery microbiota with or without pinitol in the presence of naringenin. Asterisks on bars indicate significant difference between treatments (*** $P < 0.001$).

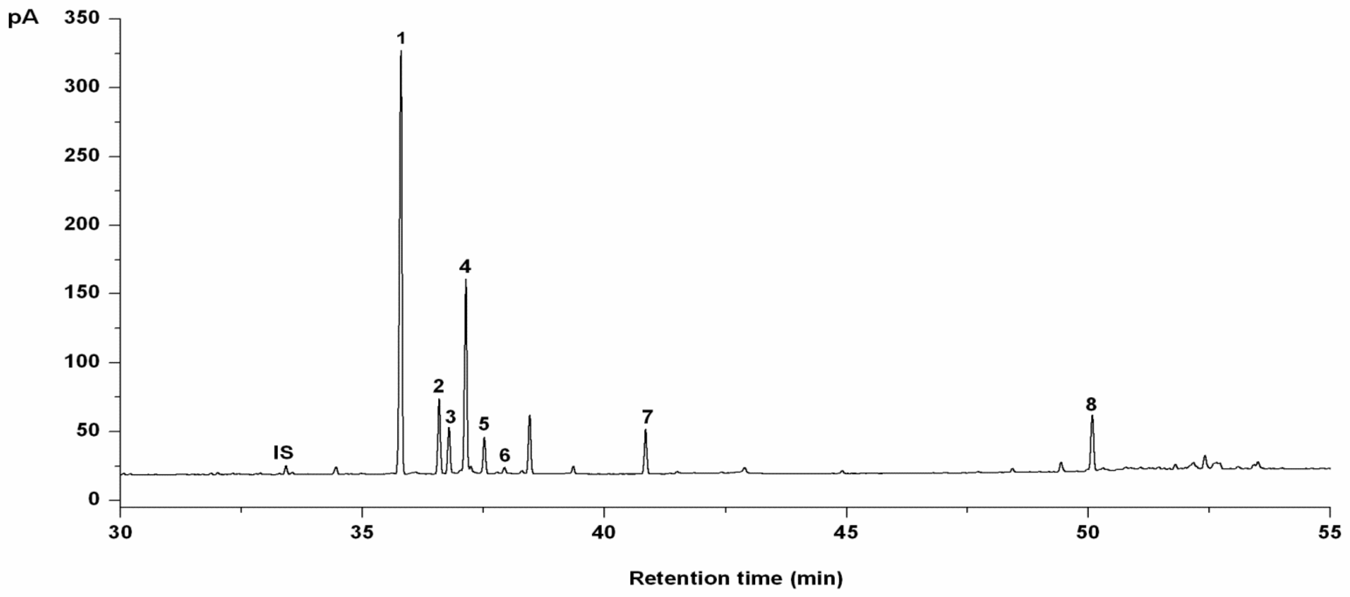

Figure S9 | GC chromatogram of soluble carbohydrates identified from healthy *P. tabuliformis* seedling phloem. Peak identification: (1) pinitol trimethylsilylate derivative (TMS); (2) fructose MEOX1 TMS; (3) fructose MEOX2 TMS; (4) glucose MEOX1 TMS; (5) glucose MEOX2 TMS; (6) mannitol TMS; (7) *myo*-inositol TMS; (8) sucrose TMS. IS: ribitol TMS. Quantification (mean ± SE mg·g^{-1}.dw) from 15 healthy seedlings: pinitol, 47.1625 ± 2.7681; fructose, 9.8756 ± 1.8552; glucose, 18.3730 ± 1.3682; mannitol, 0.0925 ± 0.0335; *myo*-inositol, 4.3231 ± 0.3883; sucrose, 2.8449 ± 0.6447.

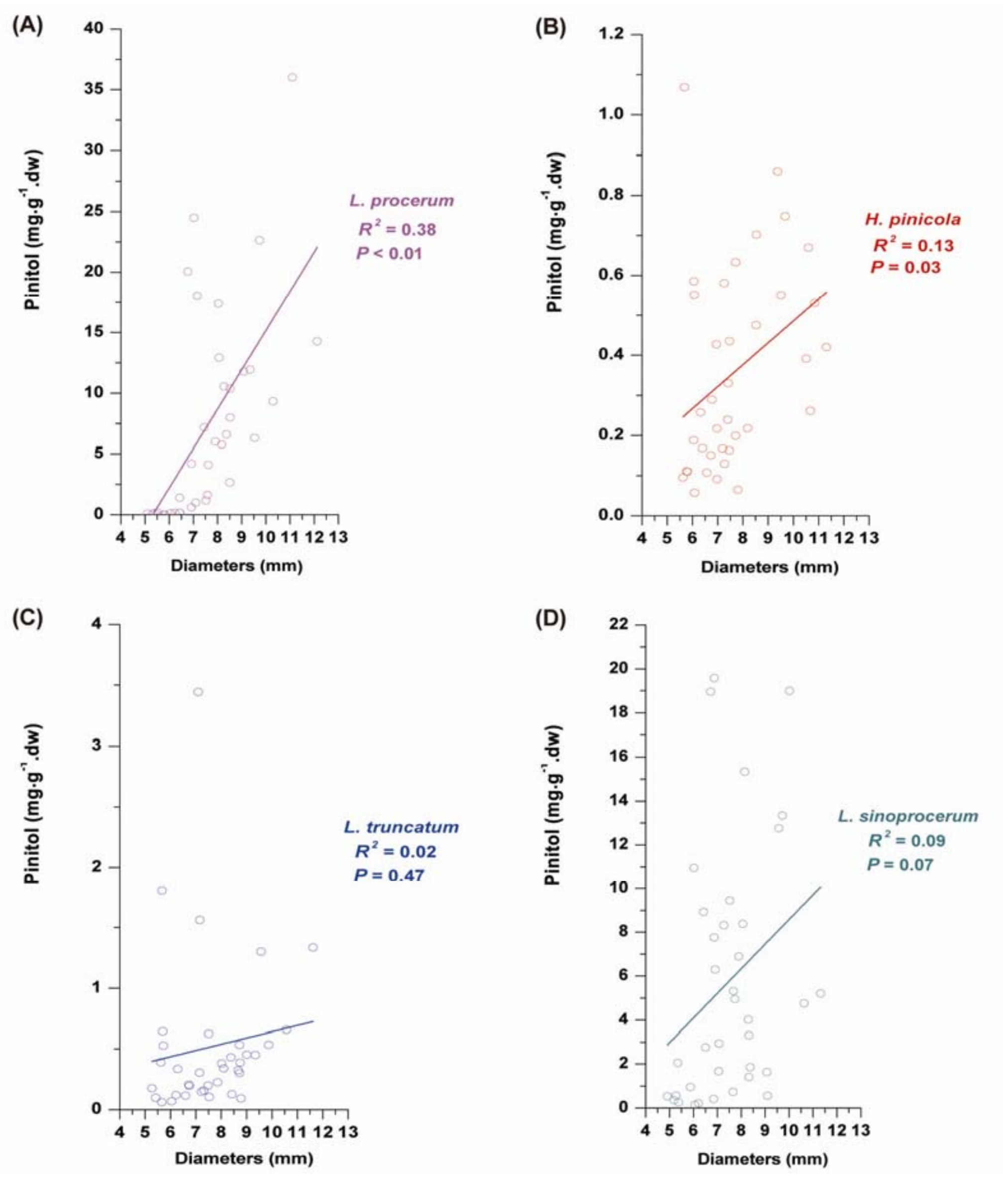

Figure S10 | Relationships between stem diameter and pinitol concentration in 4-5-yr-old *P. tabuliformis* seedlings (n = 37) inoculated with (**A**) *L. procerum*, (**B**) *H. pinicola*, (**C**) *L. truncatum* and (**D**) *L. sinoprocerum*. Diameter of *P. tabuliformis* seedling was a significant predictor of pinitol concentration for two fungal associates (*L. procerum*, $r^2 = 0.38$, $F_{1,35} = 21.62$, $P < 0.01$; *H. pinicola*, $r^2 = 0.13$, $F_{1,35} = 5.02$, $P = 0.03$), but was not significant for another two fungal associates (*L. truncatum*, $r^2 = 0.02$, $F_{1,35} = 0.53$, $P = 0.47$; *L. sinoprocerum*, $r^2 = 0.09$, $F_{1,35} = 3.58$, $P = 0.07$). The standardized slopes of regressions between *L. procerum* and each of the other three fungi were significantly different (*L. procerum*, β = 0.62, *H. pinicola*, β = 0.35; $F_{1,70} = 20.81$, $P < 0.01$; *L. truncatum*, β = 0.12; $F_{1,70} = 19.58$, $P < 0.01$; *L. sinoprocerum*, β = 0.31; $F_{1,70} = 5.32$, $P = 0.02$).

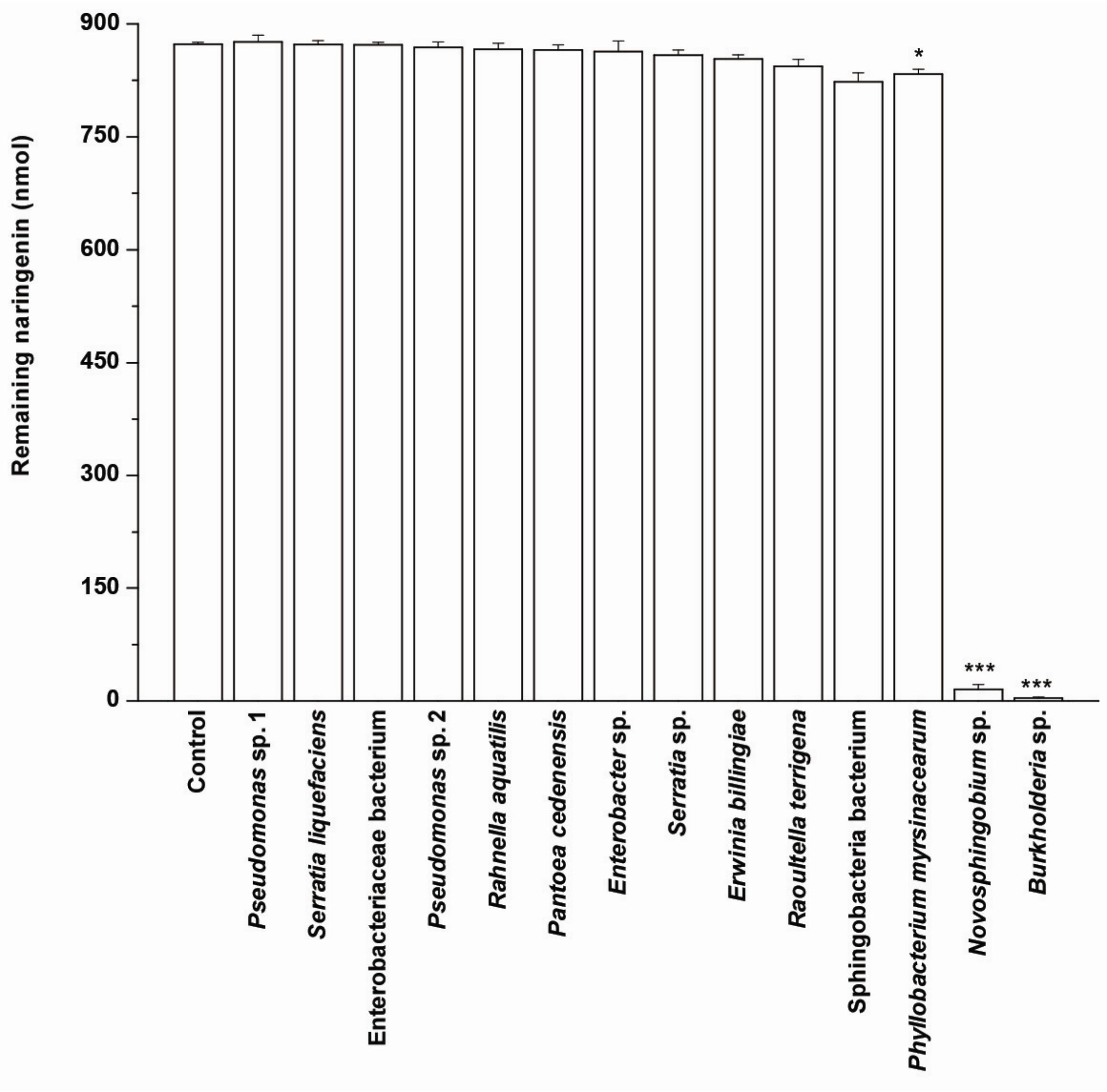

Figure S11 │ Mean quantities of remaining naringenin (+ SEM, *n* = 5) caused by bacterial species (Brown-Forsythe one-way ANOVA, $F_{14, 33.939} = 1518.565$, $P < 0.0001$). Asterisks on bars indicate significant difference between controls and bacterial species (pair-wise comparisons by Dunnett T3 test, * $P < 0.05$, *** $P < 0.001$).

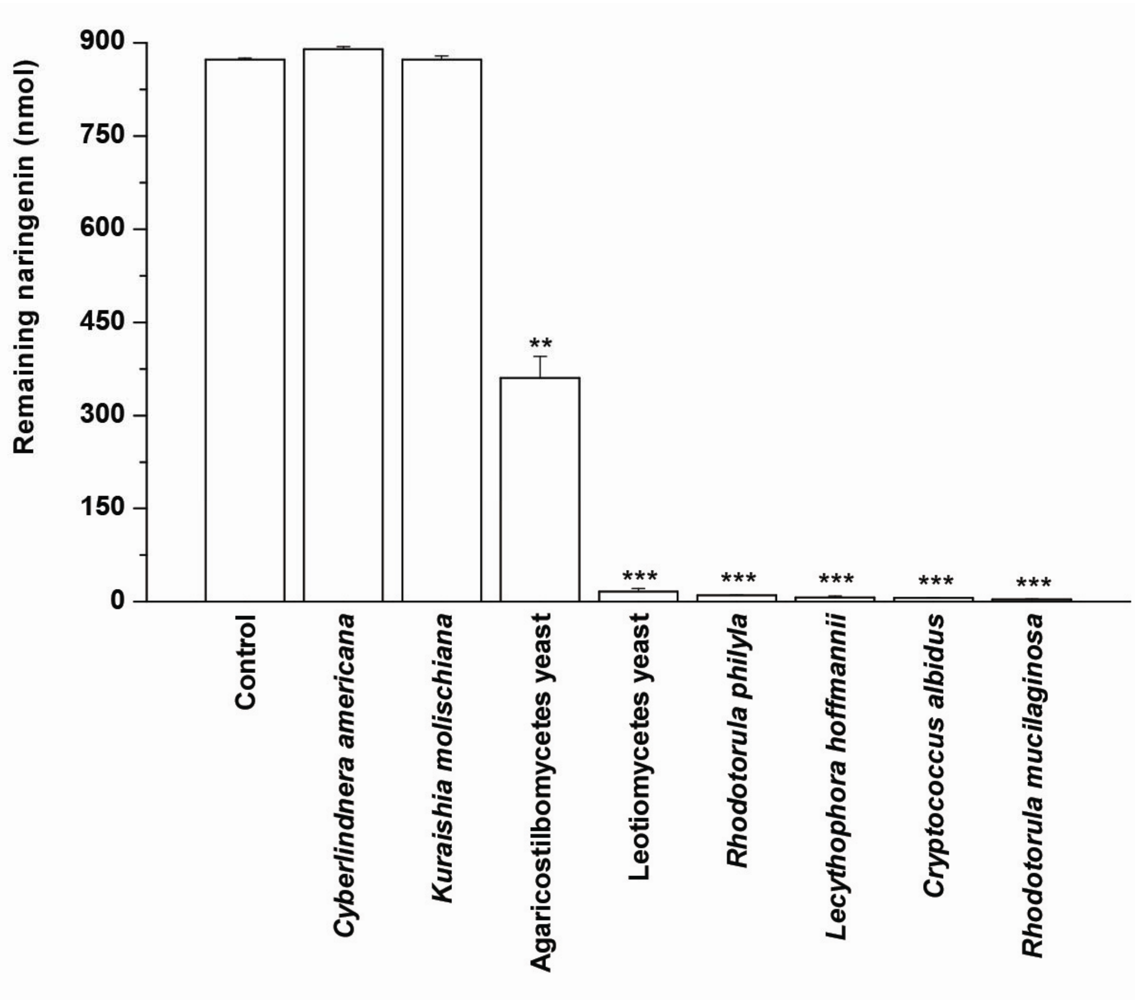

Figure S12 | Mean quantities of remaining naringenin (+ SEM, *n* = 5) caused by yeast species (Brown-Forsythe one-way ANOVA, $F_{8, 4.597}$ = 1257.845, *P* < 0.0001). Asterisks on bars indicate significant difference between controls and yeast species (pair-wise comparisons by Dunnett T3 test, ** *P* < 0.01, *** *P* < 0.001).

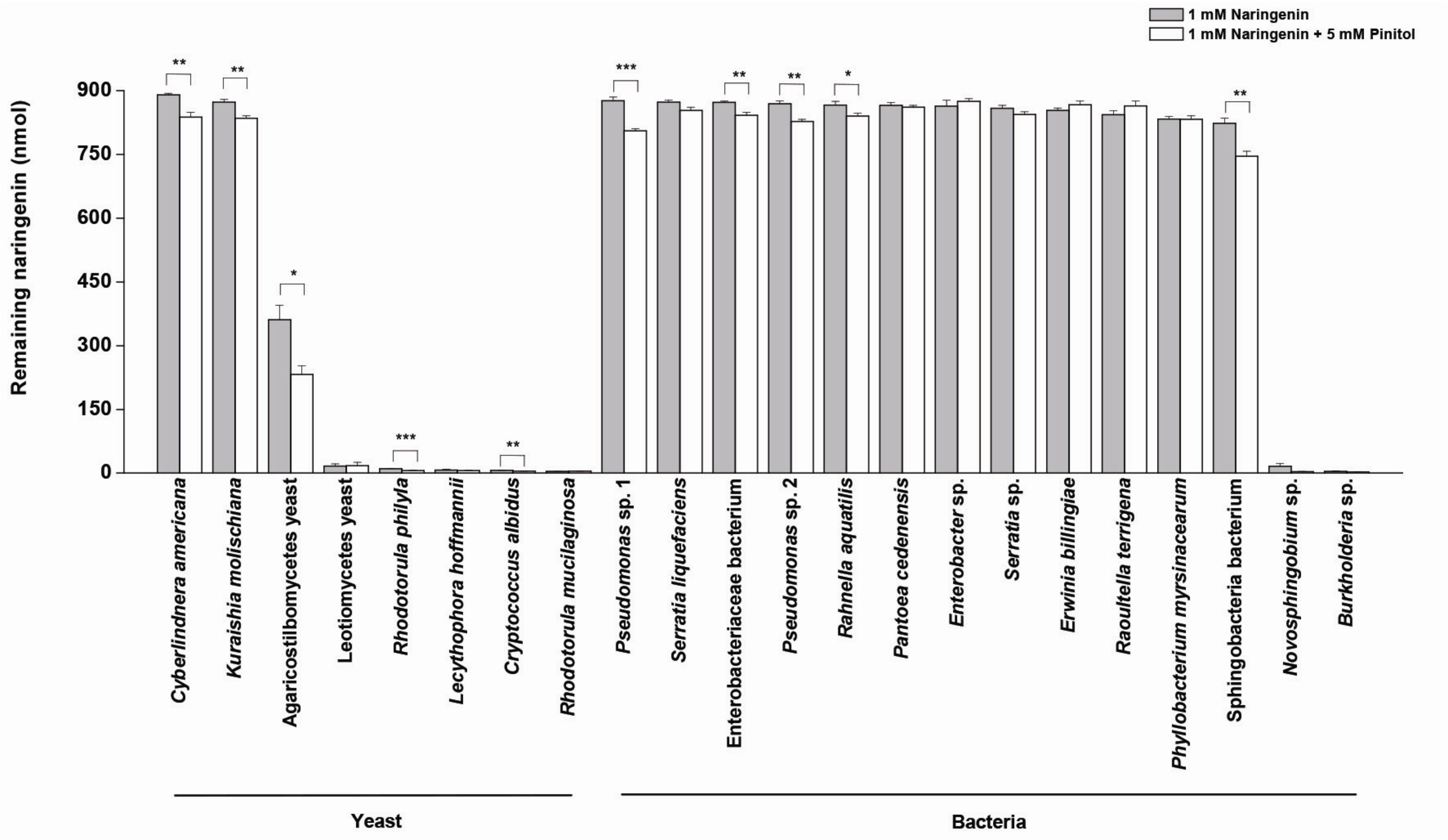
1 mM Naringenin
1 mM Naringenin + 5 mM Pinitol
Remaining naringenin (nmol)
900
750
600
450
300
150
0
Cyberlindnera americana
Kuraishia molischiana
Agaricostilbomycetes yeast
Leotiomycetes yeast
Rhodotorula philyla
Lecythophora hoffmannii
Cryptococcus albidus
Rhodotorula mucilaginosa
Pseudomonas sp. 1
Serratia liquefaciens
Enterobacteriaceae bacterium
Pseudomonas sp. 2
Rahnella aquatilis
Pantoea cedenensis
Enterobacter sp.
Serratia sp.
Erwinia billingiae
Raoultella terrigena
Phyllobacterium myrsinacearum
Sphingobacteria bacterium
Novosphingobium sp.
Burkholderia sp.
Yeast
Bacteria

Figure S13 | Comparisons of mean quantities of remaining naringenin (+ SEM, *n* = 5) caused by individual microbes with or without pinitol. Pairwise comparisons were conducted by independent *t* tests: *Cyberlindnera americana*, $t_8 = 4.350$, $P = 0.0024$; *Kuraishia molischiana*, $t_8 = 4.542$, $P = 0.0019$; Agaricostilbomycetes yeast, $t_8 = 3.183$, $P = 0.0129$; Leotiomycetes yeast, $t_8 = -0.037$, $P = 0.9713$; *Rhodotorula philyla*, $t_8 = 8.607$, $P < 0.001$; *Lecythophora hoffmannii*, $t_8 = 0.393$, $P = 0.7049$; *Cryptococcus albidus*, $t_8 = 3.817$, $P = 0.0051$; *Rhodotorula mucilaginosa*, $t_8 = -0.842$, $P = 0.4245$; *Pseudomonas* sp. 1, $t_8 = 6.695$, $P < 0.001$; *Serratia liquefaciens*, $t_8 = 2.045$, $P = 0.0751$; Enterobacteriaceae bacterium, $t_8 = 3.969$, $P = 0.0041$; *Pseudomonas* sp. 2, $t_8 = 4.614$, $P = 0.0017$; *Rahnella aquatilis*, $t_8 = 2.342$, $P = 0.0473$; *Pantoea cedenensis*, $t_8 = 0.536$, $P = 0.6067$; *Enterobacter* sp., $t_8 = -0.755$, $P = 0.4721$; *Serratia* sp., $t_8 = 1.650$, $P = 0.1375$; *Erwinia billingiae*, $t_8 = -1.218$, $P = 0.2578$; *Raoultella terrigena*, $t_8 = -1.411$, $P = 0.1958$; *Phyllobacterium myrsinacearum*, $t_8 = 0.105$, $P = 0.9191$; Sphingobacteria bacterium, $t_8 = 4.557$, $P = 0.0019$; *Novosphingobium* sp., $t_{4.037} = 1.925$, $P = 0.1259$; *Burkholderia* sp., $t_8 = 1.313$, $P = 0.2255$. * $P < 0.05$; ** $P < 0.01$; *** $P < 0.001$.

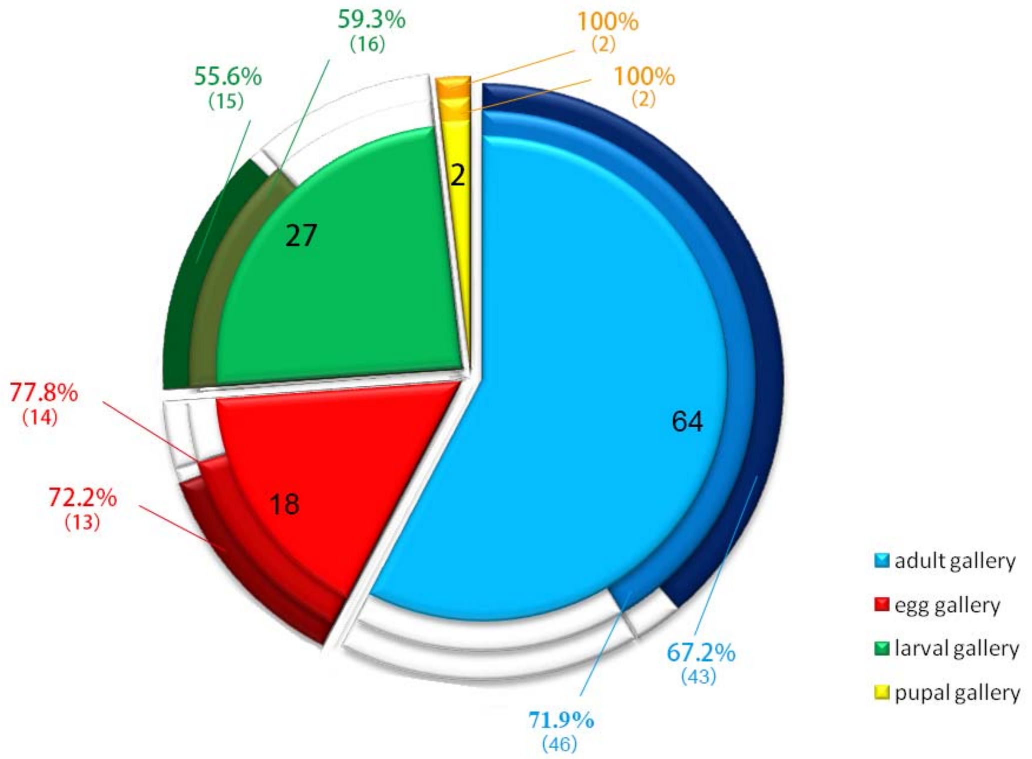

Figure S14 | Incidence of *L. procerum* and naringenin-degrading microbes in RTB galleries determined in field surveys. Inner layer: numbers of the four kinds of RTB galleries collected in the field. Middle layer: isolation ratio of *L. procerum* in each kind of RTB gallery. Outer layer: association ratio of *L. procerum* with naringenin-degrading microbiota in each kind of RTB gallery.

Tables

Table S1 | The 20 fungal species associated with RTB in China.

Fungal group	Species [a]	CN [b]	US [b]	Isolate count [c]	Reference(s) [d]
Chinese-invasive	***Leptographium procerum*** (CMW 25626)	+	+	145	1, 2, 3, 4, 5
Shared	***L. koreanum*** (CMW 29970)	+	+	9	2, 5
	Ophiostoma floccosum (CMW 25802)	+	+	17	1, 4, 5
	O. abietinum (CMW 26262)	+	+	3	1, 5
	O. ips (CMW 26255)	+	+	7	1, 6, 7
Chinese-resident	***Hyalorhinocladiella pinicola*** (CMW 25613)	+	–	2	1
	L. alethinum	+	–	6	2
	L. pini-densiflorae (CMW 25600)	+	–	19	1, 4
	L. sinoprocerum (MUCL 46352)	+	–	32	2, 4
	Leptographium sp. 1	+	–	3	4
	Leptographium sp. 2	+	–	5	4
	Leptographium sp. 3	+	–	13	4
	L. truncatum (CMW 25684)	+	–	19	1, 2, 4
	O. minus (European variety) (CMW 26254)	+	–	9	1, 4
	Ophiostoma* sp.** (O. rectangulosporium***-like) (CMW 26258)	+	–	3	1
	O. piceae	+	–	2	1
	Ophiostoma sp. 1	+	–	3	4
	Ophiostoma sp. 2	+	–	2	4
	Pesotum pini	+	–	5	2
	P. aureum	+	–	2	2

[a] Fungal species used in this study were bolded. The isolate no. was presented. CMW, Cultures of Mike Wingfield, the Forestry and Agricultural Biotechnology Institute (FABI), University of Pretoria, South Africa; MUCL, a part of the Belgian Coordinated Collections of Microorganisms (BCCM).

[b] With respect to collection localities restricted in China and in *D. valens* original region: the United States. "+" means presence of the fungal species, and "–" means absence of this fungus.

[c] The isolate count for each species was based on studies by M. Lu et al. (2009), Q. Lu et al. (2009) and Wang et al. (2013).

(Continue)

[d] References:

1. Lu M., Zhou, X. D., De Beer, Z. W., Wingfield, M. J. & Sun, J. H. Ophiostomatoid fungi associated with the invasive pine-infesting bark beetle, *Dendroctonus valens*, in China. *Fungal Divers.* **38**, 133-145 (2009).

2. Lu, Q., Decock, C., Zhang, X. Y. & Maraite, H. Ophiostomatoid fungi (Ascomycota) associated with *Pinus tabuliformis* infested by *Dendroctonus valens* (Coleoptera) in northern China and an assessment of their pathogenicity on mature trees. *Antonie Van Leeuwenhoek* **96**, 275-293 (2009).

3. Lu, M., Wingfield, M. J., Gillette, N. E., Mori, S. R. & Sun, J. H. Complex interactions among host pines and fungi vectored by an invasive bark beetle. *New Phytol.* **187**, 859-866 (2010).

4. Wang, B., Lu, M., Cheng, C., Salcedo, C. & Sun, J. Saccharide-mediated antagonistic effects of bark beetle fungal associates on larvae. *Biol. Lett.* **9**, 20120787 (2013).

5. Taerum, S. J. *et al*. Large shift in symbiont assemblage in the invasive red turpentine beetle. *PLoS ONE* **8**, e78126 (2013).

6. Owen, D. R., Lindahl Jr, K. Q., Wood, D. L. & Parmeter Jr, J. R. Pathogenicity of fungi isolated from *Dendroctonus valens*, *D. brevicomis*, and *D. ponderosae* to ponderosa pine seedlings. *Phytopathology* **77**, 631-636 (1987).

7. Klepzig, K. D., Smalley, E. B. & Raffa, K. F. *Dendroctonus valens* and *Hylastes porculus* (Coleoptera: Scolytidae): vectors of pathogenic fungi (Ophiostomatales) associated with red pine decline disease. *Great Lakes Entomol.* **28**, 81-88 (1995).

Table S2 | Mean concentrations (± SEM μg·g^{-1}.dw) of six phenolic compounds induced by mock inoculation and the three fungal groups associated with RTB in China.

Compound / Group	*ρ*-coumaric acid (Mean ± SEM)	Taxifolin (Mean ± SEM)	Ferulic acid (Mean ± SEM)	Resveratrol (Mean ± SEM)	Pinosylvin (Mean ± SEM)	Pinosylvin monomethyl ether (Mean ± SEM)
Mock inoculation	72.15 ± 18.25	149.65 ± 32.43	448.20 ± 95.70	55.83 ± 19.12	843.35 ± 312.77	129.48 ± 11.95
Chinese-invasive	97.47 ± 3.06	471.53 ± 116.94	291.60 ± 67.05	96.30 ± 14.28	1524.50 ± 174.50	712.23 ± 191.67
Shared	87.50 ± 8.48	499.05 ± 53.18	415.39 ± 29.22	74.31 ± 11.79	1514.29 ± 207.68	398.84 ± 58.03
Chinese-resident	72.26 ± 5.74	553.41 ± 68.44	348.83 ± 30.08	78.36 ± 8.87	1209.96 ± 180.10	495.17 ± 66.73
F / χ^2 value	$F_{3, 47} = 1.23$	$F_{3, 47} = 2.15$	$F_{3, 47} = 1.38$	$F_{3, 47} = 0.49$	$F_{3, 47} =0.87$	$\chi^2_{3} = 6.66$
P value	0.31	0.11	0.26	0.69	0.47	0.08

Table S3 │GenBank accession numbers of bacterial isolates from RTB gallery microbiota in China in this study [a] and similarity scores to closest type strains and ecologically related strains in NCBI according to the 16S rDNA.

**Species affiliation**	**GenBank accession number**	**Closest type strains and ecologically related strains** [b]	**Similarity (%)** [c]
**α-Proteobacteria**			
Novosphingobium sp.	KJ004493	*N. barchaimii* (JN695619)[T]	98.7
		Novosphingobium sp. (EU476057) (oral secretions of *Dendroctonus rufipennis*)	99.9
Phyllobacterium myrsinacearum	KJ004494	*Ph. myrsinacearum* (D12789)[T]	99.1
		Ph. myrsinacearum (EU420077) (root of shrubs)	100.0
**β-Proteobacteria**			
Burkholderia sp.	KJ004487-KJ004489	*B. phytofirmans* (CP001053)[T]	98.6-99.2
		B. cepacea (EU476055) (oral secretions of *D. rufipennis*)	95.9-96.6
		Burkholderia sp. (KM253245) (root of *Pinus koraiensis*)	97.7-98.8
**γ-Proteobacteria**			
Enterobacter sp.	KJ004481-KJ004482	*En. ludwigii* (AJ853891)[T]	98.2
		En. ludwigii (GQ915080) (digestive tract of *Aedes albopictus*)	99.4-99.9
Enterobacteriaceae bacterium	KJ004475	*Trabulsiella odontotermitis* (DQ453129)[T] (gut of *Odontotermes formosanus*)	97.1
		En. aerogenes (FJ811874) (gut of Mexican *D. valens*)	97.7
Erwinia billingiae	KJ004476	*Er. billingiae* (AM055711)[T]	99.7
		Er. billingiae (HQ224646) (conifer tree)	99.9
Pantoea cedenensis	KJ004464-KJ004471	*Er. typographi* (GU166291)[T] (gut of *Ips typographus*)	98.7-99.3
		Pa. cedenensis (EU476019) (oral secretions of *D. ponderosae*)	96.7-97.6
		Pa. cedenensis (FJ811867) (gut of Mexican *D. valens*)	98.9-99.6
Pseudomonas sp. 1	KJ004477	*Ps. libanensis* (AF057645)[T]	99.6
		Pseudomonas sp. 8 (KJ781918) (frass of Chinese *D. valens*)	100.0
Pseudomonas sp. 2	KJ004479	*Ps. simiae* (AJ936933)[T]	99.6
		Pseudomonas sp. 10 (KJ781921) (frass of Chinese *D. valens*)	99.4
Rahnella aquatilis	KJ004461-KJ004463	*Rah. aquatilis* (CP003244)[T]	97.5-99.6
		Rah. aquatilis (KJ781940) (gut of Chinese *D. valens*)	97.2-100.0
		Rah. aquatilis (KJ781939) (gut of Chinese *D. valens*)	98.1-100.0

Raoultella terrigena	KJ004483-KJ004485	*Rao. terrigena* (Y17658)[T]	99.4
		Rao. terrigena (EU476045) (oral secretions of *I. pini*)	96.4
		Enterobacteriaceae bacterium (KJ781880) (frass of Chinese *D. valens*)	99.6
Serratia liquefaciens	KJ004486	*S. liquefaciens* (CP006252)[T]	99.9
		S. liquefaciens (FJ811864) (gut of Mexican *D. valens*)	99.6
		S. liquefaciens (KJ781954) (Chinese *D. valens*)	99.9
Serratia sp.	KJ004472-KJ004474	*S. plymuthica* (AJ233433)[T]	97.7
		Serratia sp. (HQ538811) (*Pi. pinaster* with nematodes)	99.9
**Bacteroidetes**			
Sphingobacteria bacterium	KJ004490-KJ004492	*Flavitalea populi* (HM130561)[T]	87.5-87.9

[a] Bacterial strains were isolated using naringenin as the sole carbon source in this study.

[b] "T" indicates type strain. Ecologically related strains were given information on isolation source.

[c] Percentage of similarity of sequences was pairwise compared from Genbank.

Table S4 | GenBank accession numbers of yeast isolates from RTB gallery microbiota in China in this study [a] and similarity scores to closest type strains and ecologically related strains in NCBI according to the D1/D2 domain of LSU rDNA.

Species affiliation	GenBank accession number	Closest type strains and ecologically related strains [b]	Similarity (%) [c]
**Ascomycota**			
Cyberlindnera americana	KJ004439-KJ004452	*Cy. americana* (EF550328)[T]	99.7-99.8
		Cy. americana (KF142616) (US *D. valens*)	99.5-100.0
		Cy. americana (KF142570) (Chinese *D. valens*)	99.5-100.0
Kuraishia molischiana	KJ004454	*K. molischiana* (AY937233)[T]	99.6
		K. molischiana (KF142585) (Chinese *D. valens*)	100.0
Lecythophora hoffmannii	KJ004453	*Symbiotaphrina buchneri* (AY227718)[T] (endosymbiont of *Sitodrepa panicea*)	98.6
		L. hoffmannii (JN938881)	99.6
Leotiomycetes yeast	KJ004457	*Neobulgaria pura* (AF353608)[T]	95.6
		Leotiomycetes sp. (AB808152) (*Toxicodendron vernicifluum*)	95.7
**Basidiomycota**			
Agaricostilbomycetes yeast	KJ004458	*Bensingtonia sakaguchii* (AF363646)[T] (leaf of *Bischofia javanica*)	90.5
Cryptococcus albidus	KJ004456	*Cr. albidus* (AF335982)[T]	100.0
		Cr. albidus (FN357223) (forest of birch)	100.0
Rhodotorula mucilaginosa	KJ004455	*Rh. mucilaginosa* (AF335986)[T]	100.0
		Rh. Mucilaginosa (KF142608) (Chinese *D. valens*)	100.0
		Rh. mucilaginosa (KM246036) (endophyte of *Catucai amarelo*)	100.0
Rhodotorula philyla	KJ004459-KJ004460	*Rh. philyla* (AY512883)[T] (tunnels of *Xyleborus ferrugineus*)	100.0

[a] Yeast strains were isolated using naringenin as the sole carbon source in this study.

[b] “T” indicates type strain. Ecologically related strains were given information on isolation source.

[c] Percentage of similarity of sequences was pairwise compared from Genbank.

Table S5 | Frequencies of bacterial and yeast isolates in microbiotas of RTB galleries of different life cycle stages in China.

	Species	Isolation source (gallery type) [a]						Total [b]	% [c]
		AG	EG	YLG	OLG	PG	TAG		
Naringenin-degrading	*Cyberlindnera americana*	60	17	44	6	2	0	129 (14)	47.96%
	Rahnella aquatilis	20	13	16	1	10	4	64 (3)	23.79%
	Sphingobacteria bacterium	25	8	13	0	0	0	46 (3)	17.10%
	Burkholderia sp.	18	5	10	0	4	1	38 (3)	14.13%
	Novosphingobium sp.	5	1	6	4	11	2	29 (1)	10.78%
	Pseudomonas sp. 1	5	0	0	1	10	1	17 (1)	6.32%
	Rhodotorula mucilaginosa	5	4	4	0	0	0	13 (1)	4.83%
	Pseudomonas sp. 2	6	1	0	0	3	2	12 (1)	4.46%
	Rhodotorula philyla	5	0	2	0	0	0	7 (2)	2.60%
	Kuraishia molischiana	2	0	1	2	0	0	5 (1)	1.86%
	Agaricostilbomycetes yeast	2	0	1	0	0	0	3 (1)	1.12%
	Phyllobacterium myrsinacearum	1	1	1	0	0	0	3 (1)	1.12%
	Lecythophora hoffmannii	0	1	1	0	0	0	2 (1)	0.74%
	Enterobacteriaceae bacterium	2	0	0	0	0	0	2 (1)	0.74%
	Leotiomycetes yeast	0	1	0	0	0	0	1 (1)	0.37%
	Cryptococcus albidus	0	1	0	0	0	0	1 (1)	0.37%
Non naringenin-degrading	*Pantoea cedenensis*	11	7	10	0	2	0	30 (8)	11.15%
	Raoultella terrigena	9	2	6	0	1	1	19 (3)	7.06%
	Serratia liquefaciens	11	3	2	0	2	0	18 (1)	6.69%
	Serratia sp.	4	0	1	1	0	0	6 (3)	2.23%
	Enterobacter sp.	3	0	0	0	0	0	3 (2)	1.12%
	Erwinia billingiae	1	0	0	0	0	0	1 (1)	0.37%

[a] Abbreviation: AG, adult gallery; EG, egg gallery; YLG, younger larval (1_{st} to 3_{rd} instar) gallery; OLG, older larval gallery; PG, pupal gallery; TAG, teneral adult gallery.

[b] Numbers in parentheses indicate number of strains used for sequencing.

[c] % means frequency of individual isolate. The total number of galleries from the field survey is 269.

Table S6 | The symbiotic relationship between RTB, *L. procerum*, and naringenin-biodegrading microbiota.

Site	Tree	G [a]		LP [b]	Nab [c]	Naringenin-biodegrading microbe [d]															
						Cam	Ra	Sb	Bs	Ns	Ps_1	Rm	Ps_2	Rp	Km	Ay	Pm	Lh	Es	Ly	Cal
**Beishe observation site**	A	AG	1	-	20984.01	+	-	-	-	-	-	-	-	-	-	-	-	-	-	-	-
			2	-	10545.10	+	-	-	-	-	-	-	-	-	-	-	-	-	-	-	-
			3	-	3671.94	+	-	+	-	-	-	-	-	-	-	-	-	-	-	-	-
			4	+	5676.68	+	-	-	-	-	+	-	-	-	-	-	-	-	-	-	-
			5	-	0.00	+	+	-	-	-	-	-	-	-	-	-	-	-	-	-	-
			6	-	9905.73	-	-	-	-	-	-	-	-	-	-	-	-	-	-	-	-
			7	+	33693.68	-	-	+	-	-	+	-	-	-	-	-	-	-	-	-	-
			8	+	348.49	+	-	-	-	-	-	-	-	-	-	-	-	-	-	-	-
			9	+	3402.59	+	-	-	+	-	-	-	-	-	-	-	-	-	-	-	-
			10	+	22652.47	+	-	-	+	-	-	-	-	-	-	-	-	-	-	-	-
		EG	1	+	1826.96	+	+	-	-	-	-	-	-	-	-	-	-	-	-	-	-
			2	+	57973.03	+	-	-	-	-	-	-	-	-	-	-	-	-	-	-	-
			3	-	9110.12	+	+	-	-	-	-	-	-	-	-	-	-	-	-	-	-
			4	+	11454.83	-	-	+	-	-	-	-	-	-	-	-	-	-	-	-	-
			5	+	9185.67	+	+	-	-	-	-	-	-	-	-	-	-	-	-	-	-
		LG	1	-	2265.50	+	-	-	-	-	-	-	-	-	-	-	-	-	-	-	-
			2	-	52757.21	+	-	-	-	-	-	-	-	-	-	-	-	-	-	-	-
			3	-	30684.13	+	-	-	-	-	-	-	-	-	-	-	-	-	-	-	-
			4	-	4405.12	+	-	-	-	-	-	-	-	-	-	-	-	-	-	-	-
			5	+	2312.86	-	+	+	-	-	-	-	-	-	-	-	-	-	-	-	-
			6	+	1386.64	-	+	-	-	-	-	-	-	-	-	-	-	-	-	-	-

**Beishe observation site**	B	AG	11	+	6059.92	+	-	-	-	-	-	-	-	-	-	-	-	-	-	-	-
			12	+	8234.23	+	-	+	-	-	-	-	-	-	-	-	-	-	-	-	-
			13	+	3302.04	-	-	-	+	-	-	-	-	-	-	-	-	-	-	-	-
			14	+	17383.35	+	-	-	+	-	-	-	-	-	-	-	-	-	-	-	-
			15	+	55950.48	+	-	+	-	-	-	-	-	-	-	-	-	-	-	-	-
			16	+	0.00	+	-	+	-	-	-	-	-	-	-	-	-	-	-	-	-
			17	+	12844.31	+	+	-	-	-	-	-	-	-	-	-	-	-	-	-	-
			18	+	22044.32	+	-	-	-	-	-	-	-	-	-	-	-	-	-	-	-
			19	+	8504.01	-	-	-	-	-	-	-	-	+	-	-	-	-	-	-	-
			20	+	14028.49	-	-	+	-	-	-	-	-	-	-	-	-	-	-	-	-
			21	+	3806.33	+	+	-	-	-	-	-	-	-	-	-	-	-	-	-	-
			22	-	2375.20	+	-	-	-	-	-	-	-	-	-	-	-	-	-	-	-
		EG	6	+	953.70	+	-	-	-	-	-	-	-	-	-	-	-	-	-	-	-
			7	+	6799.11	+	-	+	-	-	-	-	-	-	-	-	-	-	-	-	-
			8	+	37135.16	-	+	-	-	+	-	-	-	-	-	-	-	-	-	-	-
			9	+	4677.43	-	-	-	-	-	-	-	-	-	-	-	-	-	-	-	-
		LG	7	-	1123.61	+	+	-	-	-	-	-	-	-	-	-	-	-	-	-	-
			8	+	0.00	+	-	-	+	-	-	-	-	-	-	-	-	-	-	-	-
			9	+	12445.51	+	+	-	-	-	-	-	-	-	-	-	-	-	-	-	-
		PG	1	+	12663.12	+	-	-	+	-	-	-	-	-	-	-	-	-	-	-	-
	C	AG	23	+	18140.64	-	-	-	-	-	-	-	-	-	-	-	-	-	-	-	-
		LG	10	+	83026.45	+	-	-	-	-	-	-	-	-	-	-	-	-	-	-	-
	D	AG	24	+	31449.69	+	-	-	-	-	-	-	-	-	-	-	-	-	-	-	-
	E	AG	25	+	9136.18	+	-	-	+	-	-	-	-	-	-	-	-	-	-	-	-
	F	AG	26	+	8845.02	+	-	-	-	-	-	-	-	-	-	-	-	-	-	-	-

**Beishe observation site**	F	AG	27	-	0.00	+	-	-	-	-	-	-	-	-	-	-	-	-	-	-	-
		AG	28	-	15977.55	+	-	+	-	-	-	-	-	-	-	-	-	-	-	-	-
	G	AG	29	+	340.04	-	-	-	-	-	-	-	+	-	-	-	-	-	-	-	-
		AG	30	-	4521.52	-	-	-	-	-	-	-	-	-	-	-	-	-	-	-	-
		AG	31	+	3434.04	-	+	+	-	-	-	-	-	-	-	-	-	-	-	-	-
		LG	11	-	0.00	+	+	-	-	-	-	-	-	-	-	-	-	-	-	-	-
		LG	12	-	5929.72	+	-	-	-	-	-	-	-	-	-	-	-	-	-	-	-
	H	AG	32	-	15672.67	-	-	-	-	-	-	-	+	-	-	-	-	-	-	-	-
		AG	33	+	4399.97	+	-	+	-	-	-	-	-	-	-	-	-	-	-	-	-
		AG	34	+	30420.53	-	-	-	+	-	-	-	-	-	-	-	-	-	-	-	-
		LG	13	+	39511.50	+	-	-	-	-	-	-	-	-	-	-	-	-	-	-	-
		LG	14	-	28204.61	+	-	+	+	-	-	-	-	-	-	-	-	-	-	-	-
		LG	15	+	6611.73	+	-	+	-	-	-	-	-	-	-	+	-	-	-	-	-
	I	AG	35	+	1506.99	-	+	+	-	-	+	-	-	-	-	-	-	-	-	-	-
		AG	36	-	0.00	+	+	-	-	-	-	-	-	-	-	-	-	-	-	-	-
		AG	37	+	14276.03	+	-	-	-	-	-	-	-	-	-	-	-	-	-	-	-
		AG	38	+	416.79	+	-	-	+	-	-	-	-	-	-	-	-	-	-	-	-
		AG	39	+	45783.94	-	-	-	-	-	+	-	-	-	-	-	-	-	-	-	-
		AG	40	+	5765.14	-	-	+	+	-	-	-	+	-	-	-	-	-	-	-	-
		EG	10	+	25785.69	-	-	-	+	-	-	-	-	-	-	-	-	-	-	-	-
		EG	11	+	10375.70	+	-	-	-	-	-	-	-	-	-	-	-	-	-	-	-
		EG	12	-	4347.46	+	+	+	-	-	-	-	-	-	-	-	-	-	-	-	-
		LG	16	-	86921.03	+	-	-	+	+	-	-	-	-	-	-	-	-	-	-	-
		LG	17	+	642.54	-	-	-	-	-	-	-	-	-	-	-	-	-	-	-	-
	J	AG	41	-	3445.16	-	-	-	-	-	-	-	-	+	-	-	-	-	-	-	-

**Beishe observation site**	J	AG	42	-	0.00	+	-	-	-	-	-	-	-	-	-	-	-	-	-	-	-
		AG	43	-	3372.19	+	-	-	-	-	-	-	-	-	-	-	-	-	-	-	-
		EG	13	-	22833.42	+	-	-	+	-	-	-	-	-	-	-	-	-	-	-	-
		EG	14	+	482.37	-	-	-	+	-	-	-	-	-	-	-	-	-	-	-	-
		EG	15	+	0.00	-	-	-	-	-	-	-	-	-	-	-	-	-	-	+	-
		EG	16	-	4800.37	+	-	-	+	-	-	-	-	-	-	-	-	-	-	-	-
		LG	18	-	1877.58	+	-	-	-	-	-	-	-	-	-	-	-	-	-	-	-
		LG	19	+	3390.17	+	-	-	-	-	-	-	-	-	-	-	+	-	-	-	-
		LG	20	+	278.55	-	-	+	+	-	-	-	-	-	-	-	-	-	-	-	-
	K	AG	44	+	32868.21	-	-	-	-	-	-	-	-	-	-	-	-	-	-	-	-
		AG	45	+	7718.86	-	-	-	+	-	-	-	-	-	-	-	-	-	-	-	-
		AG	46	+	0.00	+	-	-	+	-	-	-	-	-	-	-	-	-	-	-	-
		AG	47	+	201.80	-	+	-	-	-	-	-	-	-	-	-	-	-	-	-	-
		AG	48	+	302.94	+	+	-	-	-	-	-	-	-	-	-	-	-	-	-	-
		AG	49	+	0.00	+	-	-	-	-	-	-	-	-	-	-	-	-	-	-	-
		EG	17	+	14960.62	-	-	+	-	-	-	-	-	-	-	-	-	-	-	-	+
		LG	21	+	8226.77	+	+	-	-	-	-	-	-	-	-	-	-	-	-	-	-
	L	AG	50	+	19068.73	-	-	-	-	-	-	-	-	-	-	+	-	-	-	-	-
	M	AG	51	+	7436.82	+	-	+	-	-	-	-	-	-	-	-	-	-	-	-	-
		AG	52	+	2874.12	+	-	+	-	-	-	-	-	-	-	-	-	-	+	-	-
		LG	22	+	12226.69	+	-	-	-	-	-	-	-	-	-	-	-	-	-	-	-
	N	AG	53	+	180273.3	+	-	+	-	-	-	-	-	-	-	-	-	-	-	-	-
		AG	54	+	2194.55	+	-	-	-	-	-	-	-	-	-	-	-	-	-	-	-
		EG	18	+	51301.18	-	+	-	-	-	-	-	-	-	-	-	-	-	-	-	-
		LG	23	+	52643.10	-	-	+	+	-	-	-	-	-	-	-	-	-	-	-	-

**Laoyagou observation site**	N	LG	24	-	3214.40	+	-	-	-	-	-	-	-	-	-	-	-	-	-	-	-
		LG	25	+	11613.74	-	+	-	-	-	-	+	-	-	-	-	-	-	-	-	-
		LG	26	+	51268.45	-	-	-	+	+	-	-	-	-	-	-	-	-	-	-	-
		PG	2	+	56329.30	-	-	-	+	-	+	-	-	-	-	-	-	-	-	-	-
	O	AG	55	+	14807.61	+	-	-	-	-	-	-	-	-	-	-	-	-	-	-	-
		AG	56	+	3233.07	-	-	-	-	-	-	-	-	-	-	-	-	-	-	-	-
	P	AG	57	+	10175.94	+	-	-	-	-	-	-	-	-	-	-	-	-	-	-	-
	Q	AG	58	+	2268.37	+	-	-	-	-	-	-	-	-	-	-	-	-	-	-	-
	R	AG	59	-	6929.58	+	-	+	-	-	-	-	-	-	-	-	-	-	-	-	-
	S	AG	60	+	2440.58	-	-	-	-	-	+	-	-	-	-	-	-	-	-	-	-
	T	AG	61	-	39634.04	+	-	-	+	-	-	-	-	-	-	-	-	-	-	-	-
	U	AG	62	-	57910.27	-	-	-	+	-	-	-	-	-	+	-	-	-	-	-	-
		AG	63	-	11804.05	+	-	-	-	-	-	-	-	-	-	-	-	-	-	-	-
	V	AG	64	+	6924.88	+	+	-	-	-	-	-	-	-	-	-	-	-	-	-	-
		LG	27	+	3207.78	+	-	-	-	-	-	-	-	-	-	-	-	-	-	-	-

[a] G: gallery identity. AG, adult gallery; EG, egg gallery; LG, larval gallery; PG, pupal gallery.

[b] LP: *Leptographium procerum*. “+” means isolated; “–” means not isolated.

[c] Nab: naringenin-biodegrading ability of individual gallery. See Fig. 4A in main text. This ability was presented as the microgram of naringenin degraded by per gram of gallery tissue in 72 h. The average dry weight of the 111 gallery tissues is 0.01 g.

[d] Abbreviations of microbial names: Cam, *Cyberlindnera americana*; Ra, *Rahnella aquatilis*; Sb, Sphingobacteria bacterium; Bs, *Burkholderia* sp.; Ns, *Novosphingobium* sp.; Ps1, *Pseudomonas* sp. 1; Rm, *Rhodotorula mucilaginosa*; Ps2, *Pseudomonas* sp. 2; Rp, *Rhodotorula philyla*; Km, *Kuraishia molischiana*; Ay, Agaricostilbomycetes yeast; Pm, *Phyllobacterium myrsinacearum*; Lh, *Lecythophora hoffmannii*; Eb, Enterobacteriaceae bacterium; Ly, Leotiomycetes yeast; Cal, *Cryptococcus albidus*. “+” means isolated; “–” means not isolated.

Methods

Experiment 1

Inoculation procedures: Fungal inoculation was conducted by making a wound on each *P. tabuliformis* seedling with a sterile 5-mm-diameter cork borer in the main stem 2 cm above the soil line to expose the cambium layer (one seedling with one inoculation point). A 5-mm-diameter plug was taken from the margin of one actively growing fungal species cultured on 2% MEA (malt extract: 7 g; agar: 7 g; distilled water: 350 ml), placed into the hole and wrapped by laboratory film ((Parafilm M; Pechiney Plastic Packaging, Chicago, IL, USA) to prevent contamination and desiccation. Mock-inoculation, where fungal induction was mimicked using a plug of 2% MEA alone (without fungi), was applied to the seedling in the same manner to serve as control.

Eleven fungal associates of RTB in China (categorized into Chinese-invasive, shared, and Chinese-resident groups; Supplementary Table S1 online) were inoculated on seedlings (n = 3 to 5 per treatment) following procedures described above. After three weeks, control tissues and treatment tissues with infected areas (between 5 mm above and below inoculation point) were excised from seedlings for chemical extraction. Fungal isolates were re-isolated from the inoculation areas to confirm that there had been no cross-contamination following methods described[1].

Phenolic compounds extraction: Before extraction, the phloem tissue (about 50 mg FW) was ground to powder in liquid N and washed with pentane (Sigma) to remove the resinous compounds, as described[2]. Soluble phenolics, cell wall-bound

phenolics and lignin were then extracted following the procedures described[3] with slight modification. Briefly, soluble phenolics were extracted in 500 µl HPLC-grade methanol (JK Chemical Corporation, Beijing) twice for 24 h at 4 ºC and the supernatants were combined, filtered through 0.45-µm Teflon syringe filters and stored at -20 ºC until analysis by HPLC as described below. Pellets from soluble phenolics procedures were then used for cell wall-bound phenolics and lignin extraction following detailed steps[3].

Chemical identification: HPLC analysis was conducted on Agilent 1100 series equipped with photodiode array detection and a scanning fluorescent detection. An Agilent HC-C18 column (particle size 5 µm, 4.6 mm × 250 mm) was carried out for chromatographic separation. Injection volume, parameters of DAD and FLD, and wavelengths for phenolic detection were as used by the described method[3], while the binary mobile phase system and linear gradient were operated with some modification. The water phase used here was: 995 ml ddH_2O, 5 ml acetic acid, 0.25 g ammonium acetate; and the organic phase was: 995 ml methanol, 5 ml acetic acid, 0.25 g ammonium acetate. The linear gradient [cumulative run time (min), flow rate (ml/min), % water phase] was changed as following: 2.0, 1.0, 100; 4.0, 1.0, 90; 29.0, 1.0, 60; 47.0, 1.75, 0.0; 48.0, 1.75, 0.0; 49.0, 1.0, 0.0; 55.0, 1.0, 0.0; 56.0, 1.0, 100; post run: 5 min.

Peak identification of soluble and cell-wall bound phenolics were conducted by comparison of UV spectral characteristics and retention times with the following standards: *p*-coumaric acid (Acros, USA), taxifolin (Sigma), ferulic acid (Apollo,

USA), resveratrol (Sigma), naringenin (Sigma), pinosylvin (Phytolab, Germany) and pinosylvin monomethyl ether (Phytolab, Germany). We further evaluated these peaks by co-injection tests with their corresponding standard compounds. The Agilent ChemStation software was used for data acquisition and peak areas of these phenolics were quantified by standard calibration curves.

Statistics: The responses of the seven phenolic compounds among fungal groups as well as response of naringenin among the eleven fungal species were tested using one-way ANOVAs followed by Bonferroni approach for pair-wise comparisons. For all one-way ANOVA analyses, we tested the normal distribution (Kolomogorov-Simirnov test) and homogeneity (Levene's test) of the variances of the responses for each treatment. Where normality and/or equal variance were not assumed, we performed nonparametric Kruskal-Wallis one-way ANOVAs, followed by pair-wise comparisons by Mann-Whitney *U* tests. We used SPSS 20 (SPSS Inc., Chicago, IL, USA) for the statistical procedures.

Experiment 2

We inoculated 4-5-yr-old *P. tabuliformis* seedlings with one of the four fungal species, including the Chinese-invasive *L. procerum* and three Chinese-resident *H. pinicola*, *L. truncatum* and *L. sinoprocerum* following inoculation procedures as described above. Seven to ten seedlings per treatment were uprooted at 3, 6, 9, 12, 18 and 24 d after inoculation. Phloem from 5mm above and below the inoculation points with distinct necrotic areas formation was excised and flash-frozen in liquid nitrogen for chemical extraction. Mock-inoculation seedlings at each time point were

sampled in the same manner. Phenolic compounds extraction and identification were followed procedures as described in Experiment 1. The polymeric phenolic compound, lignin, was detected by spectrophotometer (Beckman Coulter, USA) and quantified by standard calibration curves with five different concentrations of lignin (TCI, Japan) according to the methods described before[4].

Statistics: The responses of the eight phenolic compounds were tested using two-way ANOVAs (time and isolate as fixed factors). As we sampled tissues on independent seedlings at each time point, we assumed the responses of phenolic compounds to be independent. We then tested difference in concentrations of phenolic compounds among isolates at each time point using one-way ANOVA followed by Bonferroni approach for pair-wise comparisons when isolate in two-way ANOVA was significant. For all ANOVA analyses, we tested the normal distribution (Kolomogorov-Simirnov test) and homogeneity (Levene's test) of the variances of the responses for each treatment. Where normality and/or equal variance were not assumed, we performed nonparametric Kruskal-Wallis one-way ANOVAs, followed by pair-wise comparisons by Mann-Whitney *U* tests using Bonferroni correction to adjust the probability ($\alpha = 0.05/10 = 0.005$). We used SPSS 20 (SPSS Inc., Chicago, IL, USA) for the statistical procedures.

Experiment 3

P. tabuliformis seedlings with ascending stem diameters across pine ages (1-2- to 4-5-yr-old seedlings) were inoculated with each of the four fungal species (*L. procerum*, *H. pinicola*, *L. truncatum* and *L. sinoprocerum*) on the main stems 2 cm

above the soil line following procedures as described in Experiment 1. Eight to ten seedlings per treatment in each pine age were uprooted at 24 d after inoculation. We excised the phloem tissues near the inoculating points and flash-freeze them in liquid nitrogen for naringenin extraction and quantification following procedures as described in Experiment 1.

Statistics: We used linear regression analysis to model bivariate relationships between stem diameter of pine seedling and concentration of naringenin induced by each of the four fungal associates. Diameter of pine seedling was the predictor variable in each analysis. Further comparisons of regression coefficients between each of Chinese-resident fungi and the Chinese-invasive *L. procerum* were conducted by analysis of covariance. Here, fungal associate inoculation was the factor variable (each of Chinese-resident fungi vs. *L. procerum*) and diameter was a covariate. A significant interaction term between fungal associate inoculation and diameter indicated that the slopes of the two relationships differed significantly. We used SAS PROC REG for the statistical analyses.

Experiment 4

The boring performance of female RTB to naringenin was evaluated by tunnelling length test according to the protocol[5] with minor modifications. Phloem medium was prepared as described[6] and then molten media were transferred into a transparent glass tube (4 mm inner diameter, 50 mm in length) with enough space left at the open end for one beetle. Six levels of concentrations of naringenin (50 $\mu g \cdot g^{-1}$, 100 $\mu g \cdot g^{-1}$, 200 $\mu g \cdot g^{-1}$, 500 $\mu g \cdot g^{-1}$, 1000 $\mu g \cdot g^{-1}$ and 2000 $\mu g \cdot g^{-1}$ of diet dry

weight) were applied. Naringenin was dissolved in ethyl acetate and mixed into molten media to yield the appropriate percentages by dry mass (micrograms of naringenin per grams of dry phloem media). Ethyl acetate alone was used as control. In addition, we assessed the suitability of this solvent for evaluating in female RTB performances. No differences were found in female performances between solvent and solventless treatment (t = -1.679, df = 40, P = 0.101 for tunnelling length; t = -1.355, df = 19.802, P = 0.191 for body weight change after 6 h).

Beetles were surface-sterilized with commercial bleach, ethanol and distilled water [10 : 10 : 80 (vol : vol)], kept in a climate-controlled incubator (25 ± 1 ºC, RH = 70 %, darkness) and starved for 24 h. The beetle was inserted head-down into each tube, and then sealed with parafilm. Eighteen replicates were applied to treatments and tunnelling lengths were measured at half-hour intervals (30 min, 60 min, 90 min, 120 min and 150 min). In a separate trial, initial body weight of each beetle was recorded before insertion into tube and the ultimate body weight was recorded after 6 h to test the effect of naringenin on body weight. Twenty six to thirty replicates were applied to treatments. Short observation duration for assessing effects of phenolic compounds on performances of bark beetles was known to be sufficient, with reasonable explanations by previous study[5].

Statistics: We analyzed differences in female tunnelling length by linear mixed-effects modelling including treatment (concentrations of naringenin) and time as fixed factors, in which time accounted for repeated measures per individual. Individual was treated as random effect. Time was also treated as a covariate. We

chose Toeplitz as the best covariance structure for the model after comparisons with other structures using the Akaike information criterion (ACI). The non-significant interaction was excluded from the final model ($F_{6,\ 148.366} = 1.873$, $P = 0.089$). When appropriate, *post-hoc* comparisons between times, or between treatments within a particular time, were performed using univariate F-tests with Bonferroni adjustment for multiple comparisons built into the MIXED procedure. Data for female body weight change was analyzed by one-way ANOVA. Female body weight change was rank-transformed prior to analysis to equalize variances. Significant differences between treatments were further compared by the Student-Newman-Keuls *post-hoc* test (SNK-test). We used SPSS 20 for the statistical procedures.

Experiment 5

Larvae were surface-sterilized, and then cultivated on phloem media containing four antibiotics (0.5 % of tetracycline, penicillin, streptomycin and nystatin) for one week to eradicate associated microbes to the greatest extent possible. Sterilized larvae were placed onto ordinary phloem media for another week for detection of possible microbes and exclusion of lethargic larvae. We demonstrated that, after 12 days, naringenin concentrations in phloem media cannot be significantly decreased (50 $\mu g \cdot g^{-1}$; one-sample T test; mean ± S.E = 47.91 ± 3.15, $t = -0.663$, df = 12, $P = 0.520$; 2000 $\mu g \cdot g^{-1}$; one-sample T test; mean ± S.E = 1822.86 ± 90.06, $t = -1.967$, df = 12, $P = 0.073$).

A generalized randomized complete block design was set up to test whether higher concentrations of naringenin significantly decreased larval body weight in

one no-choice bioassay. Phloem media amended with six levels of concentrations of naringenin were prepared as described in Experiment 4, and then poured into 35-mm-diameter Petri dishes. Ethyl acetate alone was used as control. After solidification of medium, we made a hole at the centre of each Petri dish with a sterile 5 mm cork borer. Initial weight of each larva was recorded and placed into each hole. After 6 h, larvae were reweighed. Ethyl acetate has no effect on RTB larvae either (t = 0.451, df = 34, P = 0.655 for body weight change after 6 h).

In another no-choice bioassay, we measured feeding areas of larvae on phloem media amended with different concentrations of naringenin to evaluate whether higher concentrations of naringenin significantly decreased larval feeding areas. Phloem media (phloem powder 8 g; bacto-agar 2 g; distilled water 60 ml) amended with six levels of concentrations of naringenin (50 $\mu g \cdot g^{-1}$, 100 $\mu g \cdot g^{-1}$, 200 $\mu g \cdot g^{-1}$, 500 $\mu g \cdot g^{-1}$, 1000 $\mu g \cdot g^{-1}$ and 2000 $\mu g \cdot g^{-1}$ of diet dry weight) were applied here. One milliliter of phloem media was pipetted into a 35-mm-diameter Petri dish and evenly covered the bottom in a very thin layer, allowed to keep in a laminar flow cabinet overnight before use. One larva was placed in each Petri dish, with twenty two replicates in each treatment. After 6 h, larvae were removed from Petri dishes and the feeding areas, which were easily visible, were photographed and measured using software Image J (National Institutes of Health, Bethesda, Maryland, USA).

In two-choice bioassays, diet plugs amended with six levels of concentrations of naringenin (50 $\mu g \cdot g^{-1}$, 100 $\mu g \cdot g^{-1}$, 200 $\mu g \cdot g^{-1}$, 500 $\mu g \cdot g^{-1}$, 1000 $\mu g \cdot g^{-1}$ and 2000 $\mu g \cdot g^{-1}$ of diet dry weight) and solvent alone were made by a 1.5 cm diameter cork

borer from phloem media. Each 35 mm Petri dish contained one diet plug with only solvent and the other with naringenin. One larva was placed equidistant from the two plugs as previously described[7]. Petri dishes were kept in constant in darkness and larvae were allowed to choose diet plugs for 6 h. It was found that 6 h was sufficient for larvae to choose one plug over another. Larvae were presumed to have chosen a plug if they had fed exclusively around that plug, been boring into or already in that plug. Sixty replicates were applied in each treatment.

Long term effects of naringenin on boring rate and survival of RTB larvae were tested on phloem media amended with solvent and two levels of concentrations of naringenin (50 $\mu g \cdot g^{-1}$ and 2000 $\mu g \cdot g^{-1}$ of diet dry weight). Each larva was placed in a 5 mm hole at the centre of phloem media in a 35-mm-diameter Petri dish. Forty five replicates of each treatment were recorded daily to see if they were alive and boring into the phloem media.

Statistics: In the bioassay for larval body weight change, we partitioned larvae into four blocks by their initial body weight, and then randomized larvae into treatments in respective blocks. Treatment and block were set as fixed factors in a two-way ANOVA. The non-significant interaction between treatment and block was excluded from this model ($F_{18, 106} = 0.935$, $P = 0.539$), and when appropriate, we further compared means for treatment and block by LSD test. In the bioassay for larval feeding area, we performed nonparametric Kruskal-Wallis one-way ANOVA, followed by pair-wise comparisons by Mann-Whitney *U* tests using Bonferroni correction to adjust the probability ($\alpha = 0.05/21 = 0.00238$). In the two-choice

bioassay, the Chi-squared tests with the null hypothesis of equal expectation were used to compare responses of RTB larvae to diets with solvent alone and to diets with solvent plus naringenin. For ANOVA analyses, we tested the normal distribution (normality diagnostics) and homogeneity (Levene's test) of the variances. The above statistical analyses were performed by SPSS 20. The survival and boring rate of RTB larvae were calculated by Kaplan-Meier survival analysis[8]. Comparisons between survival curves were further tested by Log Rank (Mantel-Cox) method. We used GraphPad Prism 6 for the statistical analyses (GraphPad Prism Software, Inc., San Diego, CA).

Experiment 6

Four fungal isolates (*L. procerum*, *H. pinicola*, *L. truncatum* and *L. sinoprocerum*) were cultured on 2% malt-extract agar. The malt-extract agars were amended with naringenin by dissolving it in ethyl acetate and adding to the molten agar to yield the appropriate percentage by dry mass of MEA media. Ethyl acetate was set as control. No differences were found in fungal radial growth rates between solvent and solventless treatment (*L. procerum*: $t < 0.0001$, df = 6, $P = 1.000$; *H. pinicola*: $t = -1.155$, df = 10, $P = 0.275$; *L. truncatum*: $t = -0.475$, df = 10, $P = 0.645$; *L. sinoprocerum*: $t = -0.953$, df = 10, $P = 0.363$). Five mm fungal plugs were taken from the margin of actively growing fungal cultures and then placed on the centre of plates of 90 mm 2% MEA media, ensuring that aerial mycelia were in contact with media. Four or six replicate plates of each isolate in treatments were incubated in the dark at 25 ºC. Colony diameters on each Petri dish were measured daily along two

perpendicular lines after incubation for 2 days. When the fungus reached the edges of the Petri dish, we ended the assay and calculated average growth rate of each fungus under treatments with control and six levels of concentrations of naringenin (50 $\mu g \cdot g^{-1}$, 100 $\mu g \cdot g^{-1}$, 200 $\mu g \cdot g^{-1}$, 500 $\mu g \cdot g^{-1}$, 1000 $\mu g \cdot g^{-1}$ and 2000 $\mu g \cdot g^{-1}$ of MEA dry weight).

Statistics: In this bioassay, data were analyzed by one-way ANOVAs with treatment as a fixed factor, followed by Bonferroni *post hoc* tests for pair-wise comparisons. For ANOVA analyses, we tested the normal distribution (normality diagnostics) and homogeneity (Levene's test) of the variances. When data did not conform to equal variance, we performed one-way Brown-Forsythe's ANOVA[9], followed by pair-wise comparisons using Dunnett's T3 test [10].

Experiment 7

We collected 111 galleries including 64 adult galleries, 18 egg galleries and 27 larval galleries plus 2 pupal galleries, from a natural forest of infested *P. tabuliformis* at Tunlanchuan Forest Station in July 2012. A portion of each gallery tissue was excised and used in this experiment, while other portions of these galleries were distributed to other experiments with specific statements below. Gallery tissue was partitioned into two parts (for 6 h and 72 h, respectively), macerated in 0.5 ml 10% PBS buffer, ultrasonicated for 30 s and vortexed at medium speed for another 10 s to suspend putative microbial cells as crude extract.

Inorganic culture solution containing naringenin as sole carbon source was prepared. It consisted of (based on 200 ml): K_2HPO_4 0.07 g, KH_2PO_4 0.054 g, NaCl

0.058 g, NH_4Cl 0.106 g in 180 ml ultrapure water; $MgSO_4 \cdot 7H_2O$ 0.04 g, $CaCl_2 \cdot 2H_2O$ 0.015 g, trace-element solution[11] in 20 ml ultrapure water. The two solutions were autoclaved separately and mixed after cooling, resulting a PH 6.50 solution. 0.2 ml $FeSO_4 \cdot 7H_2O$ was added from filter-sterilized stock solutions (10 g/L). Naringenin was dissolved in 1 M NaOH and filter-sterilized before adding. The PH value of the ultimate solution was 7.40 containing 1 mM naringenin.

Quantitative measurement of naringenin-biodegrading activity of RTB galleries was performed by HPLC. The whole crude extract of each gallery tissue (~0.5 ml) was incubated in 1 ml of inorganic culture solution containing 1 mM naringenin at 30 ºC with shaking (150 rpm) in a glass test tube sealed by silica gel stopple. After 6 h and 72 h of incubation, the reaction was stopped by adding an equal volume of methanol[12]. This mixed solution was rapidly desiccated in vacuum by a concentrator (Eppendorf, Germany) and re-dissolved in 1 ml of methanol. After centrifugation at 10,000 rpm for 5 min, the organic supernatant containing remaining naringenin was transferred to a 2 ml vial (Agilent, USA) and stored at -20 ºC until analyzed by HPLC. Healthy phloem from twelve mature *P. tabuliformis* were set as control and treated the same way as galleries. Initial quantities of naringenin (actual value) were average values obtained by detecting ten 1 ml of 1 mM naringenin solution without any healthy phloem or galleries after 6 h and 72 h. Degrading activity of individual gallery or healthy phloem tissue was shown as microgram of reduced naringenin (subtract remaining quantity of naringenin caused by gallery or healthy phloem from initial quantity of naringenin) divided by gram of dry weight of gallery or healthy

phloem tissue. HPLC system that was applied here was identical to Experiment 1.

To further determine that this degrading activity was conferred by microbial symbionts distributed in RTB galleries, we used another 17 galleries to test this hypothesis. Gallery tissue was soaked in 1 ml of 10% PBS buffer followed by subsequent procedures as shown before, and then 0.4 ml of the crude extract was filtered through a sterile 0.22 μm (pore size) syringe-like filter before the extract was added into 1ml of 1 mM naringenin solution. To contrast, another 0.4 ml of the extract from the same sample was directly added into 1ml of 1 mM naringenin solution. After 72 h, remaining quantity of naringenin re-dissolved in 1 ml methanol was detected by HPLC following procedures described in Experiment 1.

To test the effect of naringenin on growth of gallery microbiota, 20 RTB galleries were used for this experiment. Inorganic culture solutions containing 5 mM pinitol with and without 1 mM naringenin were prepared. Pinitol is a common component in stems of conifers[13-15] and a main soluble carbohydrate in *P. tabuliformis* seedling phloem (Supplementary Fig. S9 online). Crude extract (40 μl) from the same gallery was added into 1 ml of those two kinds of solutions, respectively. After 72 h of incubation, we stopped the reaction using methanol. Pellets were first washed in methanol, followed by three rinses in sterile ddH_2O, and then re-suspended in 1 ml sterile ddH_2O for detection at OD_{600} by a microplate reader (Thermo, USA).

Statistics: For evaluation of naringenin-biodegrading activity of each category of RTB gallery tissue, the degrading activity of healthy phloem was deemed as the

control. We then performed nonparametric Kruskal-Wallis one-way ANOVA, followed by pair-wise comparisons by Mann-Whitney *U* tests using Bonferroni correction to adjust the probability ($\alpha = 0.05/6 = 0.0083$) since no data for 6 h or 72 h conformed normality and equal variance. To compare remaining quantity of naringenin caused by crude extracts with or without filtration and growth of gallery microbiota in pinitol with or without naringenin, we used the parametric paired-samples T test.

Experiment 8

In August 2012, 4-5-yr-old *P. tabuliformis* seedlings were inoculated with one of the four fungal species, *L. procerum*, *H. pinicola*, *L. truncatum* and *L. sinoprocerum* or 2% MEA plugs alone following approaches described in Experiment 1. Seven to ten seedlings per treatment were uprooted at 3 d, 6 d, 9 d, 12 d, 18 d, and 24 d after inoculation. We sampled the necrotic phloem tissues between 5 mm above and below the inoculation points. Samples were flash-frozen by liquid nitrogen and stored at -80 ºC.

Carbohydrates extraction: Phloem carbohydrates were extracted and derivatized following the protocols[16] with minor modification. Phloem material (about 50 mg FW) was crushed by a pestle on micro-mortar in liquid nitrogen, and then put into a 2 ml tube for further homogenization by an electronic tissue-ruptor in 500 µl of methanol containing 20 µl of ribitol (0.2 mg/ml in stock) added as internal quantitative standard. The tube was heated at 70 ºC for 15 min and mixed 3 times during incubation. After centrifugation for 10 min at 14,000 g, the supernatant was

transferred to a new 2 ml tube. Then 250 μl chloroform and 500 μl ddH_2O were sequentially added. After fractionation of non-polar metabolites into chloroform, 200 μl of polar phase was dried under vacuum and then derivatized with methoxyamine hydrochloride and MSTFA sequentially. The derivatized extract was mixed with 500 μl isooctane and 300 μl ddH_2O.

Chemical identification: Carbohydrates dissolved in isooctane were quantitatively analyzed by an Agilent 7890A GC equipped with a flame ionization detector (FID) (Agilent Technology, USA). 1 μl of sample was injected at 230 ºC with hydrogen carrier gas with flow rate set to 1 ml/min. Chromatography was performed on a HP-5 capillary column (60 m×0.25 mm×0.25μm). The temperature program was isothermal at 70 ºC for 5 min, followed by a 5 ºC/min ramp to 310 ºC, and holding at this temperature for 12 min. Cooling was performed as fast as possible. The system was then temperature equilibrated at 70 ºC for 5 min before the next injection. Main carbohydrates were previously identified by GC 6890N coupled with 5973 MSD through comparing fragmentation patterns with those in a mass spectral database and those of individual standards (D-pinitol, D-fructose, D-glucose, D-mannitol, *myo*-inositol and sucrose; Sigma). We applied relative quantification to these carbohydrates based on the internal standard[15].

Statistics: Pinitol is a main soluble carbohydrate in *P. tabuliformis* phloem; we thus focused on it for further studies. The change in pinitol levels was tested using two-way ANOVA (time and isolate as fixed factors). As we sampled tissues on independent seedlings at each time point, we assumed the change of pinitol to be

independent. Levels of pinitol in pine phloem among isolates at each time point was further compared using one-way ANOVA, followed by S-N-K *post-hoc* test for pair-wise comparisons. For ANOVA analysis, we tested the normal distribution (normality diagnostics) and homogeneity (Levene's test) of the variances. When data did not conform to conditions of parametric test, we performed nonparametric Kruskal-Wallis one-way ANOVAs, followed by pair-wise comparisons by Mann-Whitney *U* tests using Bonferroni correction to adjust the probability (α = 0.05/10 = 0.005). We used SPSS 20 for the statistical procedures.

Experiment 9

To compare the naringenin biodegrading activity and the growth of gallery microbiota in a mixture of naringenin and pinitol with those in naringenin alone, two kinds of inorganic culture solution, one containing only 1 mM naringenin, the other one containing 1 mM naringenin and 5 mM pinitol, were prepared. Eighteen RTB galleries were macerated with plastic pestles in 0.5 ml 10% PBS buffer and then 40 µl of crude extract from the same gallery was transferred into 1 ml of those two kinds of solutions, respectively. After incubation for 72 h with shaking, the reaction was stopped by adding 1 ml of methanol. Using the extraction method described in Experiment 7, 1ml methanol supernatant containing remaining naringenin was obtained for analysis by HPLC using the same conditions as in Experiment 1. Lower quantities of remaining naringenin indicated greater naringenin degradation capacity. Pellets from centrifugation were kept for measurement of growth of microbiota. Pellets were washed three times by sterile ddH_2O and re-suspended in 1 ml sterile

ddH_2O for detection at OD_{600} by the microplate reader.

Statistics: We performed a nonparametric two-related samples test (Wilcoxon signed ranks test) to compare quantity of remaining naringenin caused by microbiota in culture solutions with or without pinitol. Growth of the microbiota in naringenin with or without pinitol was compared by the parametric paired-samples T test.

Extended Methods

Relationships between stem diameter and pinitol concentration of pine seedlings inoculated with RTB fungal associates

To test the effect of tree size on induction of retention of pinitol by each of the four fungal species (*L. procerum*, *H. pinicola*, *L. truncatum* and *L. sinoprocerum*), *P. tabuliformis* seedlings with ascending diameters across pine ages were inoculated following procedures as described in Experiment 3. Eight to ten seedlings per treatment in each pine age were uprooted at 24 d after inoculation. We excised the phloem tissues near the inoculation points and then flash froze them in liquid nitrogen for pinitol extraction and quantification following procedures as described in Experiment 8.

Statistics: We used linear regression analysis to model bivariate relationships between diameter of pine seedling and concentration of pinitol changed by each of four fungal associates. Diameter of pine seedling was the predictor variable in each analysis. Further comparisons of regression coefficients between each of Chinese-resident fungi and the Chinese-invasive *L. procerum* were conducted by analysis of covariance. Here, fungal associate inoculation was the factor variable (each of Chinese-resident fungi vs. *L. procerum*) and diameter was a covariate. A significant interaction term between fungal associate inoculation and diameter indicated that the slopes of the two relationships differed significantly. We used SAS PROC REG for the statistical analyses.

Isolation and identification of bacteria and yeasts from RTB galleries

Crude extracts of another portion of 111 galleries in Experiment 7 and 158 galleries including 47 adult galleries, 22 egg galleries, 47 younger larval galleries, 12 older larval galleries, 25 pupal galleries and 5 teneral adult galleries, subsequently collected from the natural forest of infested *P. tabuliformis* in September 2012, were used to screen out individual microbial species from the microbial communities quickly after field collections. For selective enrichment, 0.1 ml of each crude extract was added to 3 ml of autoclaved inorganic culture solution containing 1 mM naringenin and incubated for 5 d at 30 ºC with shaking (150 rpm). Three serial liquid enrichment cultures were performed at the same condition, and 0.2 ml of the final liquid culture was spread on 2% select agar (BBL, Cockeysville, Md.) solidified medium containing 1 mM naringenin. After 5 d incubation at 30 ºC, a single colony for each possible species of microbe was selected and streaked for three times on naringenin solid medium. Finally, separated single colonies were further examined on an optical microscope (Olympus, Japan) and re-cultured on TSA for bacteria or YMEA for yeasts before storage at 4 ºC.

To extract bacterial 16S rDNA, pure colonies of bacterial strains were transferred to tubes with 2 ml of tryptic soy broth (TSB, sigma) medium, and incubated at 30 ºC with shaking for 12 hours. After centrifugation at 13,000 rpm (15,493 g) for 1 min, bacterial cells were re-suspended in 480 µl of 50 mM EDTA buffer (pH 8.0), digested by 20 µl lysozyme (Tiangen, China) (100 mg/ml) and water-bathed at 37 ºC for 1 h, followed by protocols of DNA extraction using a blood & cell culture DNA mini kit (Qiagen, USA).

Nearly the whole length of 16S rDNA of each bacterial strain was amplified by PCR using primers 8 forward (5'-AGAGTTTGATCATGGCTCAG-3') and 1492 reverse (5'-TACGGTTACCTTGTTACGACTT-3'). A 50 µl PCR reaction system was as follows: 2 µl template, 2 µl of each primer (10 µM), 0.8 µl *Taq* polymerase (5 U/µl) (Takara, Dalian), 1.2 µl dNTP (10 mM/each), 5 µl PCR buffer (10 ×, with $MgCl_2$) and 37 µl ddH_2O. The reaction was carried out on an Eppendorf Mastercycler Gradient (Eppendorf, Germany) with PCR condition as follows: 94 ºC for 5 min; 35 cycles of 30 s at 94 ºC, 30 s at 51 ºC, and 1 min 30 s at 72 ºC; and a final extension at 72 °C for 10 min.

DNA extraction and PCR amplification of the D1/D2 domain of LSU rDNA of each yeast strain were performed according to steps described above with minor modification: Primer sets: NL1 (5'-GCATATCAATAAGCGGAGGAAAAG-3') and NL 4 (5'-GGTCCGTGTTTCAAGACGG-3'); PCR condition: 94 ºC for 5 min; 35 cycles of 30 s at 94 ºC, 30 s at 54 ºC, and 1 min 30 s at 72 ºC; and a final extension at 72 °C for 10 min.

The PCR products were used for sequencing after verification by 1% agarose gel electrophoresis. Consensus sequences were assembled in MEGA 5 with manual editions according to chromatograms visualized in Chromas. The accession numbers of sequences registered in the GenBank database were listed in Supplementary Table S3 and S4 online.

Naringenin biodegradation by bacteria and yeasts isolated from RTB galleries

Representative strains of each species identified in the above experiment were

transferred to tubes with 2 ml of TSB (for bacterial strains) or YMEB (0.3% yeast extract, 0.3% malt extract, 1% glucose, 0.5% tryptone and 2% agar; for yeast strains), and incubated at 30 ºC for 24 h. We adjusted the OD_{600} values of culture solutions for all strains to 0.5, and then 10 μl of each adjusted solution was added into 1 ml inorganic culture solutions containing 1 mM naringenin with and without 5 mM pinitol. In addition, 1 ml of 1 mM naringenin alone was set as control. Five replicates for control and strains (treatments) were applied. After 30 ºC incubation for 72 h with shaking (150 rmp), remaining naringenin was extracted from each culture solution following steps in Experiment 7, re-dissolved in 1 ml methanol and then analyzed by HPLC using identical conditions as in Experiment 1.

Statistics: Quantity of remaining naringenin among control and treatments were performed by one-way Brown-Forsythe's ANOVA for yeast group and bacterial group, respectively, allowing unequal variances. *Post hoc* pair-wise comparisons were done using Dunnett's T3 test. Quantity of remaining naringenin caused by individual strain with or without pinitol was compared by the parametric independent-samples T test.

Field survey for the Chinese-invasive fungus and its association with naringenin-biodegrading microbes in RTB galleries

The remaining portions of 111 RTB galleries in Experiment 7 were performed for isolation and identification of *L. procerum*. Each gallery phloem was cut into small pieces using sterile surgical scissors, and then directly placed on the surface of media selective for ophiostomatoid fungi (2% MEA amended with 0.05%

cycloheximide and 0.04% streptomycin) in Petri dishes, which were sealed with parafilm and incubated at 25 ºC. We checked daily and carefully transferred presumed hyphae of *L. procerum* onto new media to obtain a pure culture when observed. Further identification of isolated fungal strains was carried out by comparing morphological characteristics with the stock isolate (CMW 25626) on 2% malt extract agar medium (2 g malt extract, 2 g agar, and 100 ml double distilled water), 2% agar medium (2 g agar, 100 ml double distilled water), pine twig medium (2 g malt extract, 2 g agar medium, 100 ml double distilled water and one pine twig), and 1.5% oat agar medium (1.5 g oat powder, 2 g agar and 100 ml double distilled water). The combined information of naringenin-biodegrading microbes in each gallery with the isolation results of *L. procerum* here, led us to investigate the closeness of their association in the field.

References

1. Eckhardt, L. G., Jones, J. P. & Klepzig, K. D. Pathogenicity of *Leptographium* species associated with loblolly pine decline. *Plant Dis.* **88**, 1174-1178 (2004).
2. Lieutier, F. *et al*. Changes in phenolic metabolites of Scots-pine phloem induced by *Ophiostoma brunneo-ciliatum*, a bark-beetle-associated fungus. *Eur. J. Plant Pathol.* **26**, 145-158 (1996).
3. Bonello, P. & Blodgett, J. T. *Pinus nigra-Sphaeropsis sapinea* as a model pathosystem to investigate local and systemic effects of fungal infection of pines. *Physiol. Mol. Plant Pathol.* **63**, 249-261 (2003).
4. Bonello, P. & Pearce, R. B. Biochemical defence responses in primary roots of Scots pine challenged *in vitro* with *Cylindrocarpon destructans*. *Plant Pathol.* **42**, 203-211 (1993).
5. Faccoli, M. & Schlyter, F. Conifer phenolic resistance markers are bark beetle antifeedant semiochemicals. *Agric. For. Entomol.* **9**, 237-245 (2007).
6. Wang, B., Lu, M., Cheng, C., Salcedo, C. & Sun, J. Saccharide-mediated antagonistic effects of bark beetle fungal associates on larvae. *Biol. Lett.* **9**, 20120787 (2013).
7. Cipollini, D., Stevenson, R., Enright, S., Eyles, A. & Bonello, P. Phenolic metabolites in leaves of the invasive shrub, *Lonicera maackii*, and their potential phytotoxic and anti-herbivore effects. *J. Chem. Ecol.* **34**, 144-152 (2008).
8. Vilcinskas, A., Stoecker, K., Schmidtberg, H., Röhrich, C. R. & Vogel, H. Invasive harlequin ladybird carries biological weapons against native competitors.

Science **340**, 862-863 (2013).

9. Myers, J. L. & Well, A. D. *Research Design and Statistical Analysis* (Lawrence Erlbaum Associates, Mahwah, NJ, 2003).

10. Dunnett, C. W. Pairwise multiple comparisons in the unequal variance case. *J. Am. Stat. Assoc.* **75,** 796-800 (1980).

11. Shelton, D. R. & Tiedje, J. M. General method for determining anaerobic biodegradation potential. *Appl. Environ. Microbiol.* **47,** 850-857 (1984).

12. Kikuchi, Y. *et al*. Symbiont-mediated insecticide resistance. *Proc. Natl. Acad. Sci. USA.* **109,** 8618-8622 (2012).

13. Hoch, G., Richter, A. & Körner, C. Non-structural carbon compounds in temperate forest trees. *Plant Cell Environ.* **26,** 1067-1081 (2003).

14. Lehto, T., Lavola, A., Julkunen-Tiitto, R. & Aphalo, P. J. Boron retranslocation in Scots pine and Norway spruce. *Tree Physiol.* **24,** 1011-1017 (2004).

15. Yeh, T. F., Morris, C. R., Goldfarb, B., Chang, H. M. & Kadla, J. F. Utilization of polar metabolite profiling in the comparison of juvenile wood and compression wood in loblolly pine (*Pinus taeda*). *Tree Physiol.* **26,** 1497-1503 (2006).

16. Lisec, J., Schauer, N., Kopka, J., Willmitzer, L. & Fernie, A. R. Gas chromatography mass spectrometry-based metabolite profiling in plants. *Nat. Protoc.* **1**, 387-396 (2006).
